# Centromere Binding and a Conserved Role in Chromosome Stability for SUMO-Dependent Ubiquitin Ligases

**DOI:** 10.1371/journal.pone.0065628

**Published:** 2013-06-13

**Authors:** Loes A. L. van de Pasch, Antony J. Miles, Wilco Nijenhuis, Nathalie A. C. H. Brabers, Dik van Leenen, Philip Lijnzaad, Markus K. Brown, Jimmy Ouellet, Yves Barral, Geert J. P. L. Kops, Frank C. P. Holstege

**Affiliations:** 1 Molecular Cancer Research, University Medical Centre Utrecht, Universiteitsweg 100, Utrecht, The Netherlands; 2 Institute of Biochemistry, Swiss Federal Institute of Technology, ETH-Hönggerberg, Zürich, Switzerland; Newcastle University, United Kingdom

## Abstract

The *Saccharomyces cerevisiae* Slx5/8 complex is the founding member of a recently defined class of SUMO-targeted ubiquitin ligases (STUbLs). Slx5/8 has been implicated in genome stability and transcription, but the precise contribution is unclear. To characterise Slx5/8 function, we determined genome-wide changes in gene expression upon loss of either subunit. The majority of mRNA changes are part of a general stress response, also exhibited by mutants of other genome integrity pathways and therefore indicative of an indirect effect on transcription. Genome-wide binding analysis reveals a uniquely centromeric location for Slx5. Detailed phenotype analyses of *slx5*Δ and *slx8*Δ mutants show severe mitotic defects that include aneuploidy, spindle mispositioning, fish hooks and aberrant spindle kinetics. This is associated with accumulation of the PP2A regulatory subunit Rts1 at centromeres prior to entry into anaphase. Knockdown of the human STUbL orthologue RNF4 also results in chromosome segregation errors due to chromosome bridges. The study shows that STUbLs have a conserved role in maintenance of chromosome stability and links SUMO-dependent ubiquitination to a centromere-specific function during mitosis.

## Introduction

Accurate inheritance of chromosomes during each cell division is crucial for cell survival. Genome instability is disadvantageous and directly associated with many diseases including cancer [1]. Cells have a wide variety of regulatory mechanisms that monitor the fidelity of DNA replication and mitosis. At a molecular level, control of genome stability is an intrinsic process that depends on proper posttranslational modification of many proteins. This includes ubiquitination and sumoylation. Ubiquitin and SUMO (small ubiquitin-like modifier) are small peptides that can be covalently attached to substrates through a three-step enzymatic cascade that activates (E1), conjugates (E2) and ligates (E3) the peptide to a substrate [2]–[4]. In *S. cerevisiae*, the genes *SLX5* and *SLX8* encode the heterodimeric protein complex Slx5/8, which is required for maintenance of genome integrity in yeast [5], [6]. Biochemical characterisation of the complex has revealed that Slx5/8 is a SUMO-dependent ubiquitin E3 ligase [7], [8]. Both Slx5 and Slx8 have a C-terminal zinc finger RING domain, commonly found in many ubiquitin E3 ligases [9]. Slx5/8 also has multiple SUMO-interaction motifs, which confers a unique ability to ubiquitinate and degrade sumoylated proteins [7], [8]. This indicates that SUMO-targeted ubiquitin ligases (STUbLs) control the turnover of sumoylated proteins via ubiquitin-dependent protein degradation to ensure appropriate cellular levels of sumoylated proteins [10].

The cellular role of STUbLs is less well characterised. *SLX5* and *SLX8* were originally identified in a screen for genes that are synthetic lethal with deletion of *SGS1*, a DNA helicase of the RecQ family, indicating a role for Slx5/8 in genome stability [5]. Inactivation of STUbLs leads to a broad spectrum of genome instability phenotypes in *S. cerevisiae* and *S. pombe.* These include a strong cell cycle delay, DNA damage checkpoint activation, sensitivity to genotoxic stress, gross chromosomal rearrangements and increased rates of DNA mutation and recombination [6], [11]–[15]. The Slx5/8 complex resides at sites of DNA damage and replication, and contributes to DNA repair by relocating double stranded DNA breaks to the nuclear pore [11], [12], [16]. How the role of Slx5/8 in genome stability ties in with its function as STUbL is unclear and other cellular roles have also been proposed. STUbL orthologues have now been identified from fission yeast (Rfp1, Rfp2, spSlx8) to human (RNF4), indicating an evolutionarily conserved and important function for ubiquitin-dependent degradation of sumoylated proteins [14], [15], [17], [18]. In humans, there is evidence that RNF4 regulates transcription with several transcription regulators identified as targets for SUMO-dependent ubiquitination [19]–[21]. In yeast, Slx5/8 has also been implicated in transcription regulation [8], [22]–[24], indicating that STUbL function may extend beyond genome stability.

To better characterise the function of STUbLs, the phenotypes of *slx5*Δ and *slx8*Δ deletion mutants in transcription and genome stability were investigated in detail. We show that changes in mRNA expression in *slx5/8* mutants are largely associated with a general stress response that is likely due to genome instability rather than a direct transcriptional defect. Determination of the genomic location of the Slx5/8 complex reveals that Slx5 locates at centromeres. Loss of *SLX5* or *SLX8* is accompanied by accumulation of Rts1 at centromeres during metaphase. Moreover, the *slx5*Δ and *slx8*Δ mutants display a variety of mitotic defects, supporting a role for Slx5/8 in chromosome stability. This is distinct from previously reported roles of Slx5/8 and gives a better insight into how genome instability arises in *slx5/8* mutants. Analysis of human RNF4 shows that the role of Slx5/8 in chromosome stability is evolutionarily conserved, further underscoring the importance of STUbL function in genome stability during mitosis.

## Results

### *Slx5*Δ and *slx8*Δ Display a General Stress Response that is Shared With Mutants of Various Genome Integrity Pathways

**Figure 1:**
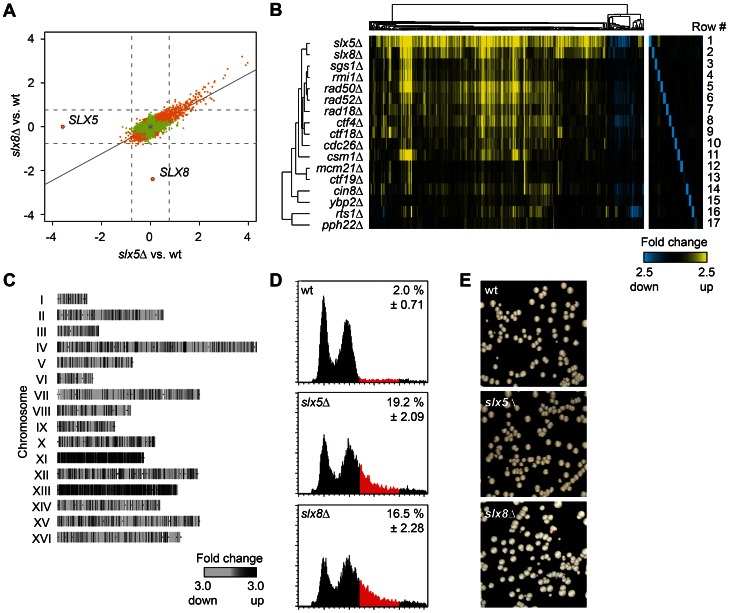
*Slx5*Δ and *slx8*Δ display a genome instability-induced stress response. (A) Scatter plot comparing the changes in mRNA expression levels in *slx5*Δ and *slx8*Δ mutants. Fold change (FC) in expression is the average of four measurements for each mutant (two independent cultures each measured twice), plotted as log_2_ values of mutant over wt. Genes changing significantly (*p*<0.05) are indicated in orange (significant in both mutants) or green (significant in one mutant). Deleted genes are indicated. Solid line indicates the regression line. Dashed lines mark a 1.7 FC threshold. (B) Heatmap and cluster diagram of the gene expression profiles of deletion mutants, showing all significant genes (FC >1.7, *p*<0.05) that change at least once in any mutant. FC expression of mutant over wt is indicated by the colour scale, with yellow for upregulation, blue for downregulation and black for no change. Deleted genes are depicted in the right-hand panel. (C) Microarray expression profile of an aneuploid *slx8*Δ mutant with duplications of chromosome XI and XIII. Genes are mapped per chromosome. The grey scale indicates FC expression in the *slx8*Δ strain versus wt. (D) Flow cytometric profiles of asynchronous populations of wt, *slx5*Δ and *slx8*Δ. Cell population with a >2N DNA content, indicated in red, is quantified (± s.d., n = 3). (E) Chromosome loss assay of wt, *slx5*Δ and *slx8*Δ cells. Red-sectoring of colonies reflects loss of the reporter chromosome. Frequency of chromosome missegration events is 0.03% (n = 1 out of 3415 colonies) for wt, 0.06% (n = 2 out of 3254, *p* = 0.247) for *slx5*Δ, and 0.11% (n = 4 out of 3555, *p* = 0.022) for *slx8*Δ. Frequencies are quantified by colony half-sector analysis and significance of the difference of mutant compared to wt was determined using the binomial test.

To investigate the role of Slx5/8 in transcription, genome-wide mRNA levels in *SLX5* and *SLX8* deletion strains were compared to wild type (wt), all grown to mid-log phase under standard growth conditions. A strong transcriptional response is observed upon *SLX5* deletion, resulting in changed expression of 321 genes (fold change (FC) >1.7, *p*<0.05, Figure 1A). The response in *slx8*Δ is quantitatively weaker (132 genes, FC >1.7, *p*<0.05), but correlates highly with *slx5*Δ (r = 0.81, Figure 1A). The similarity is also readily observed upon visual inspection of individual genes and consists largely of upregulated expression (Figure 1B; rows 1–2). The differentially expressed genes are enriched for various Gene Ontology (GO) terms (Table S1), some of which have previously also been associated with the environmental stress response in yeast [25]. The *slx5*Δ and *slx8*Δ mutant profiles were therefore compared with previously published DNA microarray datasets of various stress responses, which all share a similar expression response [25]. This reveals a significant correlation between *slx5*Δ and *slx8*Δ expression profiles and stress responses such as heat shock (r = 0.48, *p* = 2.95E-200), indicating constitutive activation of a stress response in both *slx5*Δ and *slx8*Δ under normal growth conditions.

Although Slx5/8 have previously been implicated in regulating transcription, one interpretation of the observed changes in gene expression is that these are indirectly caused by cellular stress as a consequence of the genome instability known to occur in *slx5*Δ and *slx8*Δ. To investigate this, DNA microarray expression profiles were generated for deletion mutants of various genome integrity pathways (Figure 1B). These mutants include components of the Sgs1-Rmi1-Top3 DNA helicase complex (*sgs1*Δ, *rmi1*Δ), DNA repair factors (*rad18*Δ, *rad50*Δ, *rad52*Δ), Cohesin components (*ctf4*Δ, *ctf18*Δ), a component of the anaphase promoting complex (*cdc26*Δ), a kinesin motor protein (*cin8*Δ), kinetochore-associated proteins (*csm1*Δ, *ctf19*Δ, *mcm21*Δ, *ybp2*Δ) and protein phosphatase 2A subunits (*pph22*Δ, *rts1*Δ). The transcription responses of all these mutants show a high degree of similarity to each other and to *slx5*Δ and *slx8*Δ, differing mainly in the degree of upregulation, rather than in the affected genes (Figure 1B). The differentially expressed genes are enriched in DNA binding sites for the stress response transcription factors Msn2 and Msn4 (Table S1) [26], [27]. Perturbation of several different genome integrity pathways therefore result in a similar gene expression response that is related to stress. A likely explanation for a large part of the gene expression response in *slx5*Δ and *slx8*Δ is therefore that this results indirectly from genome instability-induced stress rather than from a direct defect at the level of transcription of all these genes.

### *Slx5*Δ and *slx8*Δ Mutants are Aneuploid

The DNA microarray analyses of *slx5*Δ and *slx8*Δ also reveals a second unanticipated phenotype, the occurrence of whole chromosome aneuploidy, an example of which is shown in Figure 1C. Microarray analyses were performed on liquid cultures derived from independent colonies for each mutant. In *slx5*Δ and *slx8*Δ, aneuploidy of various chromosomes (VII, XI, XII and XIII) was observed in the form of an apparent upregulation of all genes from one or more of these chromosomes. Detection of aneuploidy in this way has been described before [28]. Note that the expression profiles shown in Figure 1A and B are the average of two independent colonies per mutant where no aneuploidy was observed. Since the detection of aneuploidy in DNA microarray experiments depends on singular events in the starting colonies, flow cytometric profiles were generated to examine the DNA content of individual cells (Figure 1D). In asynchronous cell cultures, *slx5*Δ and *slx8*Δ mutants are characterised by a large fraction of the cell population having a DNA content higher than 2N (19.2%, 16.5% and 2% in *slx5*Δ, *slx8*Δ and wt respectively). The flow cytometry therefore agrees with the aneuploidy observed in individual *slx5*Δ and *slx8*Δ microarray experiments. A colony colour assay was performed to measure the chromosome stability in *slx5*Δ and *slx8*Δ [29]. Wt, *slx5*Δ and *slx8*Δ cells, bearing the *ochre* mutation *ade2-101*, were complemented with a reporter chromosome bearing the *SUP11* gene that suppresses the red colour (Figure 1E). Chromosome missegregation events of the reporter chromosome were analyzed. Although *slx8*Δ shows a statistically significant increase of missegregation events (*p* = 0.022), this increase is only marginal and not statistically significant for *slx5*Δ (*p* = 0.247). The *slx5*Δ, *slx8*Δ aneuploidy is therefore not likely caused by chromosome loss or nondisjunction.

### Slx5 Resides at the Centromere

**Figure 2:**
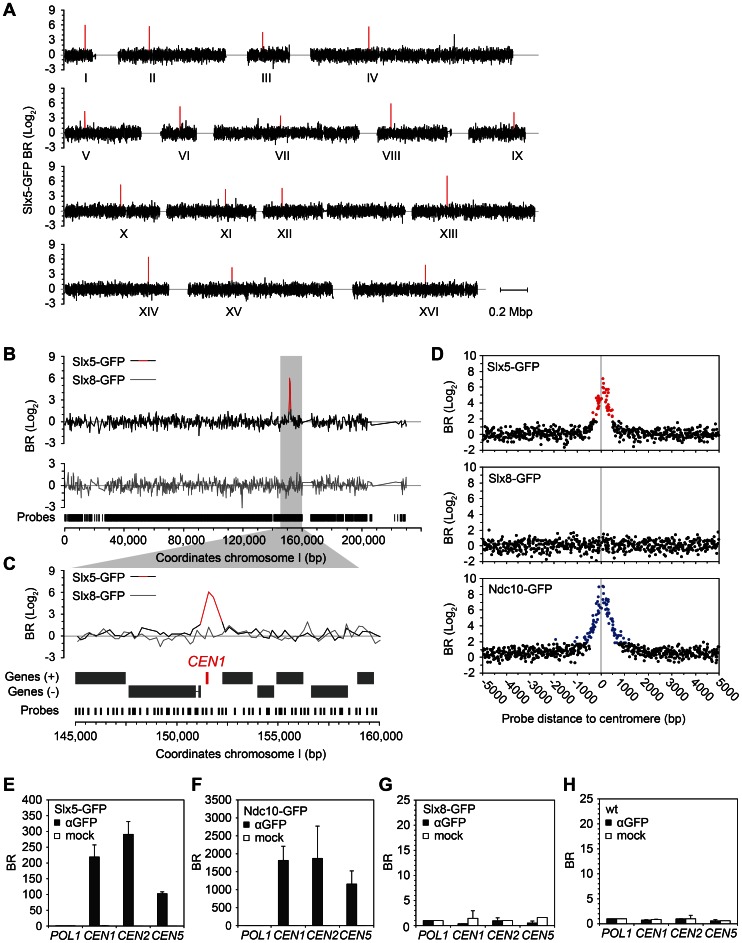
Slx5 resides at centromeres. (A) ChIP-chip analysis of Slx5. Binding ratios (BR) of individual probes are mapped to the 16 chromosomes of the *S. cerevisiae* genome. BR is expressed as log_2_ value of αGFP ChIP/input with subtraction of the mock/input signal. The positions of the centromeres are marked I to XVI. (B) Binding profile of Slx5 and Slx8 at chromosome 1. The genomic region shown in grey is magnified in (C). (D) Average centromeric binding profiles of Slx5, Slx8 and Ndc10. Signals from microarray probes are mapped relative to their position to the centromere and all probes are included that map to within 5000 bp of all 16 centromeres. Probes with a BR (log_2_) >2 are shown in red (Slx5) or blue (Ndc10). (E–H) ChIP-qPCR of Slx5 (E), Ndc10 (F), Slx8 (G) and wt (H). BRs at centromere 1, 2, and 5 are normalised to the control gene *POL1* (± s.d., n = 3).

To further elucidate the function of Slx5/8, their location on DNA was investigated by genome-wide chromatin immunoprecipitation (ChIP-chip). This was motivated by the observation that previously reported roles of Slx5/8, such as transcription [8], [22]–[24] and DNA repair [11], [12], [16], may be associated with location on DNA. Slx5 and Slx8 were C-terminally fused to GFP by genomic integration, resulting in expression at endogenous levels. ChIP-chip reveals the presence of 17 distinct binding peaks for Slx5 (Figure 2A). Strikingly, each Slx5 peak maps to a different chromosome and coincides exactly with the location of the centromere. One exception is chromosome IV, where a second smaller Slx5 peak is detected (Figure 2A). As opposed to Slx5, Slx8 did not show enrichment at centromeres. For example, whereas chromosome I shows a single centromeric Slx5 peak, we did not detect any coinciding Slx8 signal (Figure 2B and C). Other genomic locations, such as ORFs, promoters, (sub-)telomeres, ARS, or rDNA, do not show notable enrichment for Slx5 or Slx8.

Centromeres play a key role in chromosome segregation. They provide the binding site for the kinetochore, which physically connects centromeres to microtubules, allowing segregation of sister chromatids during mitosis and meiosis [30]. The centromeres of budding yeast are small (∼120 bp) and known as point centromeres. Each Slx5 peak is characterised by enriched signals on three to five consecutive microarray probes that span a centromere (e.g. Figure 2C). An average centromere binding profile was generated by mapping all (peri-)centromeric probes relative to their respective centromere (Figure 2D). Slx5 enrichment is centred on the core centromere, without global enrichment of the entire 10 kb pericentromeric region. We compared the binding profile of Slx5 to a known kinetochore component, Ndc10, which is the centromere DNA binding subunit of the kinetochore [31]. Strikingly, the binding pattern of Slx5 is equivalent to Ndc10, showing that Slx5 is preferentially located at the core centromere (Figure 2D). Centromeric location of Slx5 was further confirmed by ChIP quantitative real-time PCR. Slx5 and Ndc10 are highly enriched at all centromeres tested (Figure 2E and F), in agreement with the genome-wide experiments. Mock ChIPs, Slx8 and an untagged wt strain show no centromeric enrichment (Figure 2E–H), confirming the specific location of Slx5 to centromeres.

**Figure 3:**
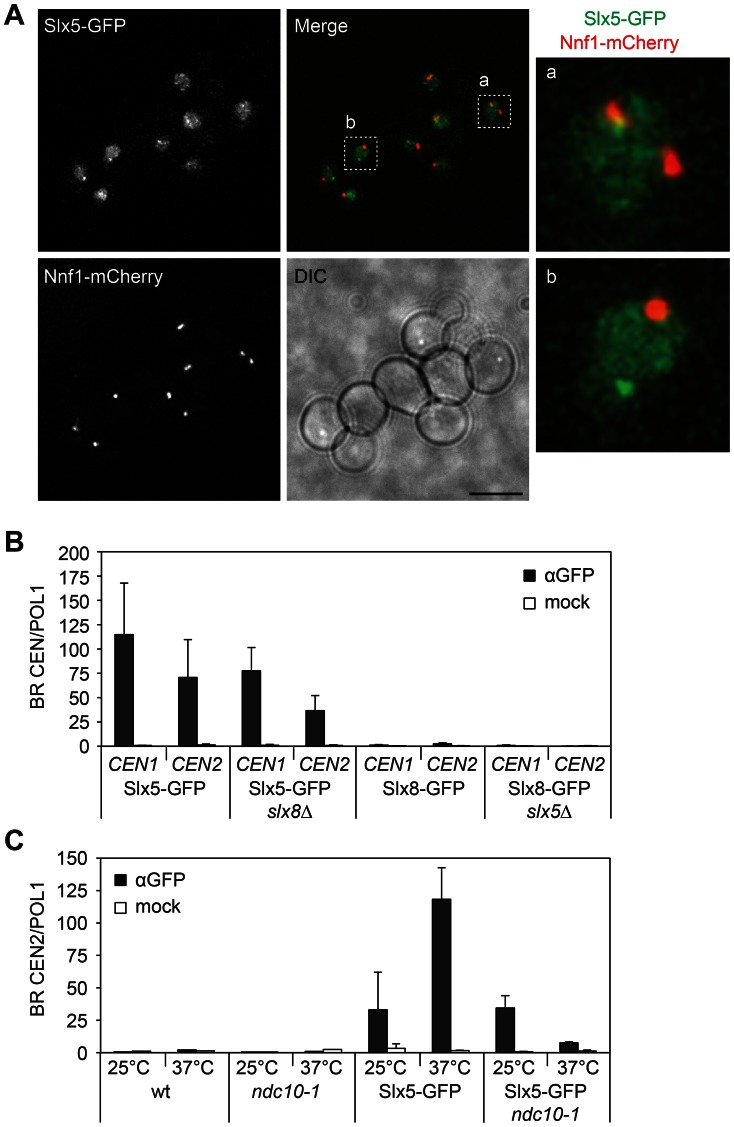
Slx5 binding to centromeres is largely kinetochore-dependent. (A) Live cell microscopy of cells coexpressing Slx5-GFP and kinetochore protein Nnf1-mCherry. Right-hand panel shows a magnification of two nuclei, indicated in the merged image. Scale bars, 5 µm. (B) ChIP-qPCR of Slx5-GFP and Slx8-GFP in wt, *slx5*Δ or *slx8*Δ strains. Binding ratios (BR) at *CEN1* and *CEN2* are represented as enrichment over the control gene *POL1* (± s.d., n = 4), based on two independent biological replicate experiments. (C–D) ChIP-qPCR of Slx5-GFP in wt and *ndc10-1* strains at permissive (25°C) and nonpermissive (37°C) temperatures. Data is represented as enrichment at *CEN2* over *POL1* (Slx5-GFP and Slx5-GFP *ndc10-1*: ± s.d., n = 2) (wt and *ndc10-1*: n = 1, no enrichment).

The subcellular localisation of Slx5 was investigated further by fluorescence microscopy (Figure 3A). Slx5 has a diffuse nuclear location with occasional subnuclear foci. This is similar to other studies, where Slx5 is shown to have a diffuse nuclear location with DNA foci at DNA repair centres [16]. We investigated whether Slx5 foci also colocalise with centromeric regions, as marked by kinetochore subunit Nnf1 (Figure 3A). Strict colocalisation of the Slx5 foci with Nnf1 was not observed. Although in rare cases Slx5 foci do overlap with the kinetochore (Figure 3A, cell a) it cannot be ruled out that they may represent cases where a DNA break is in close proximity to the centromere. This indicates that the centromeric pool of Slx5 cannot be distinguished visually by fluorescence microscopy and that it is likely part of the diffuse nuclear Slx5 pool (Figure 3A, cell b).

We next tested the dependency of the Slx5 centromeric location on other proteins by ChIP. Slx5 binding to centromeres is hardly reduced in the absence of *SLX8* (Figure 3B). This agrees with previous observations showing that Slx5 still forms nuclear foci and binds to DNA breaks without Slx8 [16]. Slx8 also remains absent from centromeres upon deletion of *SLX5* (Figure 3B). To investigate whether centromere location of Slx5 is kinetochore-dependent, a ChIP was performed in the *ndc10-1* mutant, which is defective in kinetochore assembly at a nonpermissive temperature [31]. Centromeric binding of Slx5 is nearly completely eliminated when shifting Slx5-GFP *ndc10-1* cells from 25°C to 37°C (Figure 3C). There is some residual binding, indicating that although centromere location of Slx5 is largely kinetochore-dependent, additional kinetochore-independent interactions may also be involved.

### Rts1 Accumulates at Kinetochores in *slx5*Δ and *slx8*Δ Metaphase Cells

**Figure 4:**
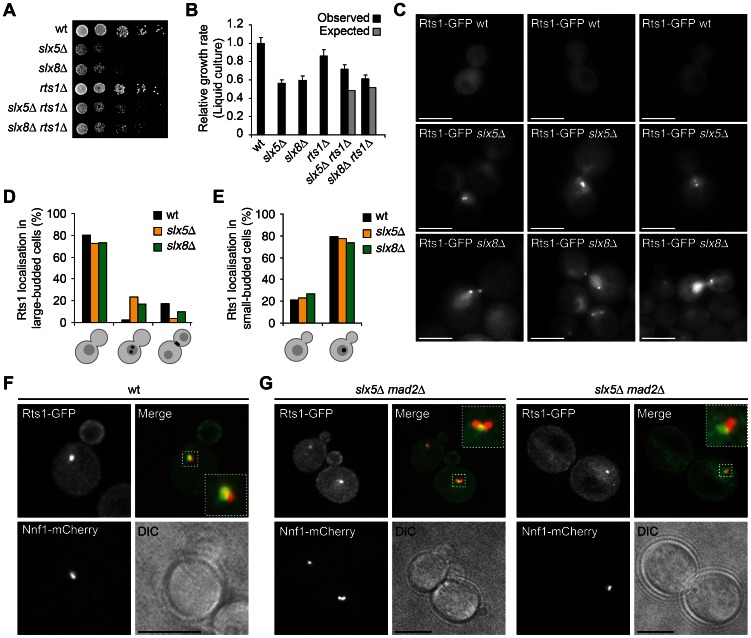
*Slx5*Δ and *slx8*Δ mutants accumulate Rts1 at kinetochores during metaphase. (A) Growth rate of cells spotted in five-fold serial dilutions on YPD plates. Images are after two days of growth at 25°C. (B) Growth rate of yeast in liquid YPD media. Relative growth rate (mutant/wt) was quantified during mid-log phase (± s.d., n = 6). Expected relative growth rates (RGR) of the double deletion mutants are calculated by multiplying the observed RGRs of the single deletion mutants. (C) Live cell microscopy of large-budded wt, *slx5*Δ and *slx8*Δ cells expressing Rts1-GFP. Nuclear Rts1 foci are detected in *slx5*Δ and *slx8*Δ, which are absent in large-budded wt cells. Scale bars, 5 µm. (D–E) Quantification of subcellular Rts1 location in wt, *slx5*Δ and *slx8*Δ cells. An asynchronous cell population (n >200 cells) was morphologically divided in small-budded (G1-S phase) and large-budded (G2/M phase) cells. Diffuse Rts1 location in nucleus and cytoplasm is schematically indicated in grey. Presence of one or two Rts1 foci is schematically indicated as black nuclear dots. Enrichment of Rts1 at the bud neck is indicated as a black bar. Rts1 foci were not detected in nonbudded cells. (F–G) Live cell fluorescence microscopy of wt (F) and *slx5*Δ *mad2*Δ cells (G), expressing Rts1-GFP and kinetochore protein Nnf1-mCherry. The small-budded wt has a normal centromeric Rts1 focus. Note that Rts1 is also enriched at the bud membrane. The left panel in (G) shows two small-budded *slx5*Δ *mad2*Δ cells with normal Rts1 foci that colocalise with kinetochores. The right panel in (G) shows a large-budded *slx5*Δ *mad2*Δ cell with an aberrant, mislocalised centromeric Rts1 focus during metaphase. Scale bars, 5 µm.

A role for Slx5/8 that is associated with a centromeric location is particularly interesting since this may better explain the genome stability defects of *slx5*Δ and *slx8*Δ mutants. Since many different regulatory pathways influence chromosome segregation, a candidate-based approach was adopted to determine factors that may be involved in the same pathway as Slx5/8, focusing in particular on potential targets. In yeast, kinetochore proteins Ndc10, Cep3 and Bir1 are sumoylated and Ndc10 interacts with Slx5 by yeast two-hybrid [32]. Changes in protein levels or subcellular location in *slx5/8* mutants were not found for any of these candidates (Figure S1). Mutations in most centromere components display either no or only weak negative synthetic genetic interactions with *slx5*Δ and *slx8*Δ [33], [34]. Positive genetic interactions are more likely indicative of gene products functioning in the same protein complex or pathway. It was therefore of interest that *RTS1*, a regulatory subunit of the PP2A phosphatase [35], was found to have positive genetic interactions with both *SLX5* and *SLX8* in a high-throughput genetic interaction map [33]. *Rts1*Δ also has a similar mRNA expression phenotype as *slx5*Δ and *slx8*Δ (Figure 1B). To confirm the genetic interaction, single and double deletion strains were generated and growth was examined on solid medium (Figure 4A) and quantified in liquid cultures (Figure 4B). The double mutants *slx5*Δ *rts1*Δ and *slx8*Δ *rts1*Δ indeed grow better than is expected from the growth of single deletion mutants, confirming the positive synthetic genetic interactions of the pairs *RTS1*-*SLX5* and *RTS1*-*SLX8*.

Rts1 is a very dynamic centromeric protein that localises to specific subcellular sites in mitotic cells in a cell cycle-dependent manner [36], [37]. Rts1 localises to kinetochores in small-budded cells, then disappears and relocates to the bud neck in large-budded cells during cytokinesis [37]. We investigated the localisation of Rts1-GFP in asynchronous wt, *slx5*Δ and *slx8*Δ cells using live cell fluorescence microscopy. Strikingly, a subpopulation of large-budded *slx5*Δ and *slx8*Δ cells are characterised by the presence of aberrant Rts1 foci (Figure 4C and D). These Rts1 foci are rarely observed in large-budded wt cells. The cells presenting these foci appear arrested in metaphase and typically have one or two Rts1 foci. The Rts1 foci in small-budded *slx5*Δ and *slx8* cells are indistinguishable from wt (Figure 4E). The foci colocalise with the kinetochore protein Nnf1 (Figure 4F and G), demonstrating that Rts1 accumulates at centromeres during metaphase in *slx5*Δ and *slx8*Δ cells. Deletion of the Spindle Assembly Checkpoint (SAC) component *MAD2* does not affect the location of Rts1 (Figure 4G), indicating that the centromeric location of Rts1 during metaphase is independent of the SAC.

**Figure 5:**
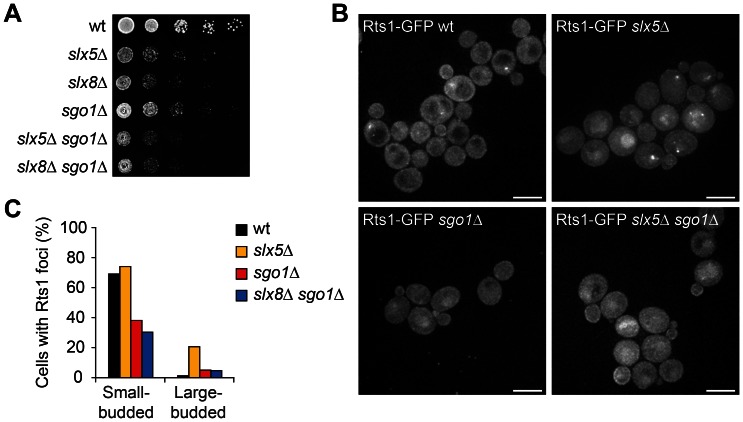
Rts1 foci are partially Shugoshin (Sgo1)-dependent. (A) Growth rate assay of cells spotted in five-fold serial dilutions on YPD plates. Images are after two days growth at 25°C. (B) Live cell fluorescence microscopy of asynchronous wt, *slx5*Δ, *sgo1*Δ and *slx5*Δ *sgo1*Δ cells expressing Rts1-GFP. Scale bars, 5 µm. (C) Quantification of Rts1 foci in cells, shown in (B). Quantification is based on an asynchronous cell population (n = 89–203), that was morphologically divided in a small-budded and large-budded cell population.

The centromeric accumulation of Rts1 in metaphase suggests the presence of a defect in *slx5/8* mutants that prevents the removal of centromeric Rts1 after recruitment during S-phase. Since the recruitment of Rts1 in meiotic cells is dependent on the centromere cohesion regulator Shugoshin (Sgo1) [38], we also investigated whether this is the case in mitotic cells. Deletion of *SGO1* results in a slow growth phenotype (Figure 5A). The Rts1 foci in *sgo1*Δ cells are less bright, indicating that Sgo1 promotes Rts1 recruitment to kinetochores in mitotic cells too (Figure 5B and C). Similarly, deletion of *SGO1* in *slx5*Δ cells results in Rts1 foci in large-budded cells that are less bright and also reduced in number (Figure 5B and C), showing that the aberrant Rts1 foci in *slx5*Δ are also partially Shugoshin-dependent.

### *Slx5*Δ and *slx8*Δ have Aberrant Spindle Positioning, Morphology and Elongation Kinetics

**Figure 6:**
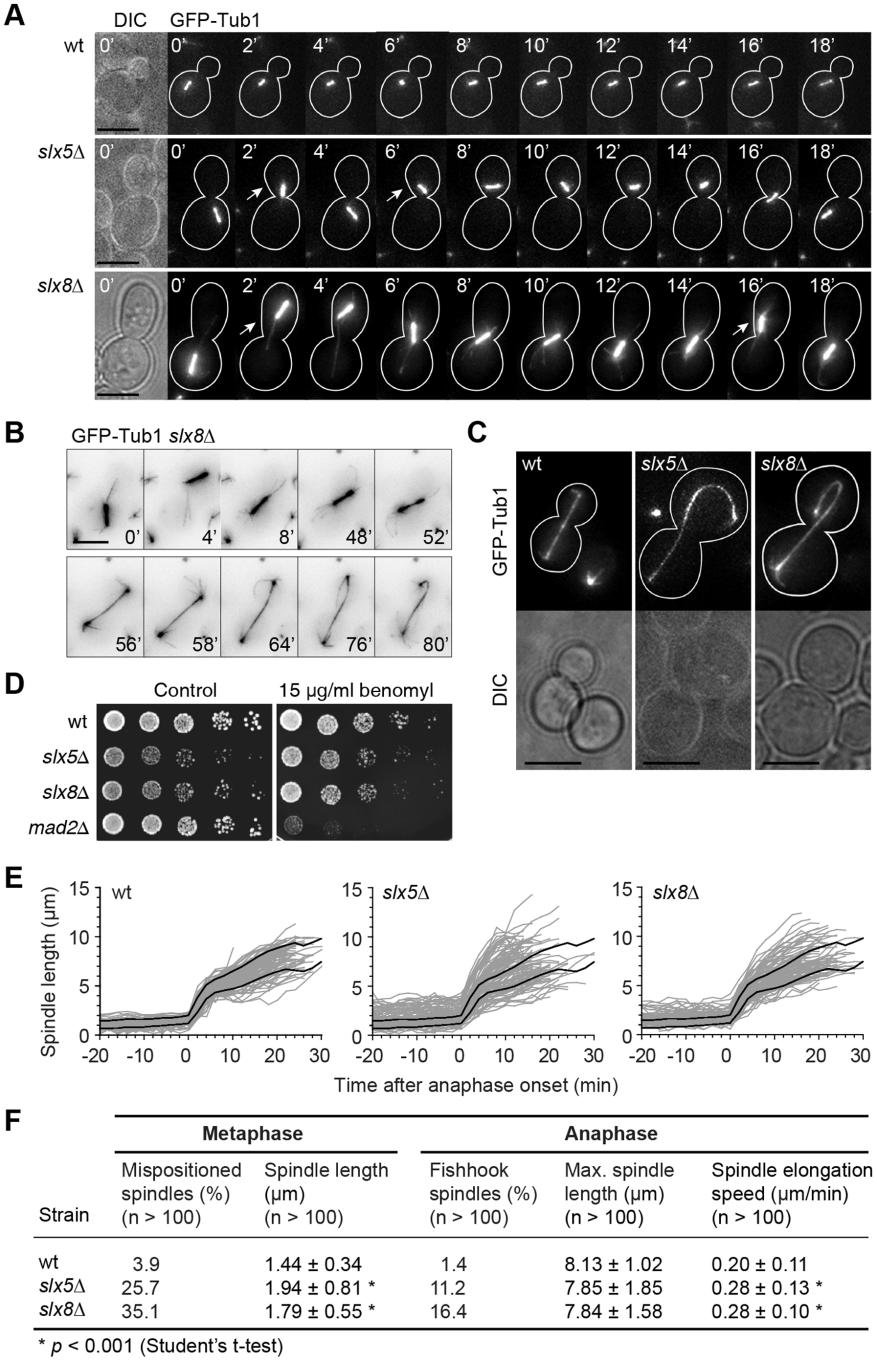
Mitotic spindle defects in *slx5*Δ and *slx8*Δ mutants. (A) Time-lapse video microscopy of wt, *slx5*Δ and *slx8*Δ cells expressing GFP-Tub1. The upper panel shows a metaphase spindle in a wt cell at 2 minute intervals. Spindle elongation is initiated at t = 18′. The two panels below show examples of aberrant positioning of metaphase spindles in *slx5*Δ and *slx8*Δ cells during a temporary metaphase arrest. Arrows indicate spindle dislocation from the bud neck. Contours of cells are marked with a white line and are based on the DIC image. Scale bars, 5 µm. (B) Example of a spindle in *slx8*Δ, followed from metaphase to late anaphase. Colours are inverted to increase visibility of the astral microtubules at the outer tips of the spindle. The cell has a prolonged metaphase (t = 0′–52′) during which the spindle dislocates into the bud (t = 4′). Entry into anaphase is initiated at t = 52′, followed by spindle extension (t = 54′–80′). Formation of a fish hook spindle is apparent during late anaphase (t = 76′–80′). Scale bar, 5 µm. (C) Examples of fish hook spindles in *slx5*Δ and *slx8*Δ and a normal elongated spindle in wt during late anaphase. Scale bars, 5 µm. (D) Benomyl sensitivity assay. Growth rate of yeast cells is measured on YPD plates complemented with benomyl or DMSO (control). Images are after two days growth at 30°C. The benomyl-sensitive SAC mutant *mad2*Δ is included as control. (E) Quantification of spindle length, defined as the distance between two spindle pole bodies in wt, *slx5*Δ and *slx8*Δ expressing Spc42-GFP. Spindle length (n >100) was quantified from 20 minutes before anaphase onset to completion of anaphase. Grey lines depict the spindle length of individual cells. Black lines represent the average wt spindle length ± s.d., which is also shown as reference in the *slx5*Δ and *slx8*Δ plots. (F) Quantifications of spindle phenotypes in wt, *slx5*Δ and *slx8*Δ during metaphase and anaphase.

In addition to the accumulation of Rts1 at the kinetochore during metaphase, a change in mitotic spindle morphology was observed in both *slx5*Δ and *slx8*Δ. The spindle morphology and dynamics were investigated in more detail using time-lapse video microscopy of asynchronous cell populations expressing GFP-Tub1 (Figure 6A). Cells were imaged at 2 minute intervals to capture the progression from metaphase into anaphase. *Slx5*Δ and *slx8*Δ arrest temporarily in mitosis as large-budded cells with short mitotic spindles and have an average mitotic delay of 80 minutes compared to wt (Figure S2A). During mitotic arrest, the cells are characterised by a spindle positioning defect in which the mitotic spindle fails to position itself stably at the bud neck (Figure 6A). Instead, the spindle oscillates heavily and frequently dislocates completely away from the bud neck, either shooting back into the mother cell or into the daughter bud. The mispositioned spindles are often accompanied by elongated astral microtubules (Figure 6A and B), which contribute to spindle positioning [39]. Around 30% of the *slx5*Δ and *slx8*Δ cells show spindle dislocation prior to entry into anaphase. This suggests that the mitotic delay in *slx5*Δ and *slx8*Δ is due to a failure in spindle positioning.

A second aberrant spindle phenotype was observed during anaphase. So-called ‘fish hook’ spindles form in 11% and 16% of the *slx5*Δ and *slx8*Δ anaphase cells respectively (Figure 6C). The formation of fish hook spindles can be a consequence of overstable microtubules and are observed in mutants of microtubule-associated proteins and kinetochore components [40]. Neither *slx5*Δ nor *slx8*Δ shows sensitivity or resistance to treatment with the microtubule-destabilising agent benomyl, suggesting that the stability of microtubules is actually normal (Figure 6D). Also the duration of anaphase in the mutants is not significantly different from wt (Figure S2B). Moreover, *slx5*Δ and *slx8*Δ do not interact genetically with SAC component *MAD2* and deletion of *MAD2* is not sufficient to overcome the mitotic arrest in *slx5*Δ and *slx8*Δ (Figure S2C and D). This indicates that microtubule-kinetochore interactions in *slx5*Δ and *slx8*Δ are normal and that Slx5/8 does not take part in the SAC signalling pathway.

The aberrant spindle morphology in *slx5*Δ and *slx8*Δ cells prompted us to investigate spindle dynamics. To analyse spindle elongation dynamics during chromosome segregation, strains expressing Spc42-GFP were imaged by time-lapse video microscopy. Spc42-GFP fluorescently labels the spindle pole bodies at the outer ends of the spindle. The spindle length was measured by determining the spindle pole body distance from metaphase until late anaphase (Figure 6E and F). The average wt spindle length in metaphase is 1.44 µm ±0.34. Upon entry into anaphase this quickly increases to a maximum length of 8.13 µm ±1.02 after ∼20 minutes. In contrast, the spindle length in *slx5*Δ and *slx8*Δ is very heterogeneous during metaphase and anaphase (Figure 6E). In metaphase, the average spindle length is 30% longer than wt, 1.94 µm ±0.81 and 1.79 µm ±0.55 for *slx5*Δ and *slx8*Δ respectively. During anaphase, the maximum spindle length measured in *slx5*Δ and *slx8*Δ is on average not significantly different from wt, but in mid-anaphase, the spindle length clearly deviates (Figure 6E and F). In *S. cerevisiae*, spindle elongation during anaphase B occurs in two phases, a quick elongation during early anaphase and a slower elongation during mid- and late anaphase [39]. Interestingly, *slx5*Δ and *slx8*Δ mutants show a continuous quick spindle elongation during mid-anaphase, whereas the wt reduces its spindle elongation speed (Figure 6E). The average spindle elongation speed was measured during mid-anaphase and is 0.28 µm/min ±0.13 and 0.28 µm/min ±0.10 for *slx5*Δ and *slx8*Δ respectively. This is 40% faster than wt, which has a spindle elongation rate of 0.20 µm/min ±0.11 (Figure 6F). The duration of anaphase in the mutants was not significantly different from wt (Figure S2B). The aberrant spindle morphology in *slx5/8* mutants is therefore associated with changes in spindle kinetics during anaphase.

### *Slx5*Δ and *slx8*Δ Phenotypes Persist in the Absence of the 2 µm Plasmid

*Slx5*Δ and *slx8*Δ mutants are known to have six-fold higher levels of extrachromosomal 2 µm plasmids compared to wt, resulting in clonal lethality [11]. We investigated the hypothesis that increased levels of native 2 µm plasmids may interfere with the spindle apparatus, resulting in defective chromosome segregation. Strains were cured of the 2 µm DNA (Cir^0^) and aneuploidy and growth rate phenotypes were examined (Figure S4A, B and C). Flow cytometry clearly indicates that *slx5*Δ Cir^0^ and *slx8*Δ Cir^0^ strains still suffer from aneuploidy (Figure S4B), which is accompanied with a reduction in growth rate compared to wt Cir^0^ strains (Figure S4C). This implies that the aneuploidy and growth phenotypes of *slx5*Δ Cir^+^ and *slx8*Δ Cir^+^ are not merely a result of overload of 2 µm DNA. The phenotypes are slightly reduced in severity compared to the Cir^+^ mutant strains, indicating that the presence of 2 µm DNA does aggravate the *slx5*Δ and *slx8*Δ phenotypes. As a final check, we also investigated the effect of 2 µm DNA on spindle dynamics in *slx8*Δ and wt strains (Figure S4D and E). As expected, *slx8*Δ Cir^0^ displays a similar defect in spindle dynamics as *slx8*Δ Cir^+^ strains. This again supports the conclusion that the presence of 2 µm DNA is not the main factor responsible for the mitotic defects of *slx5*Δ and *slx8*Δ.

### Loss of hRNF4 Results in Chromosome Missegregation Due to Chromosome Bridges

**Figure 7:**
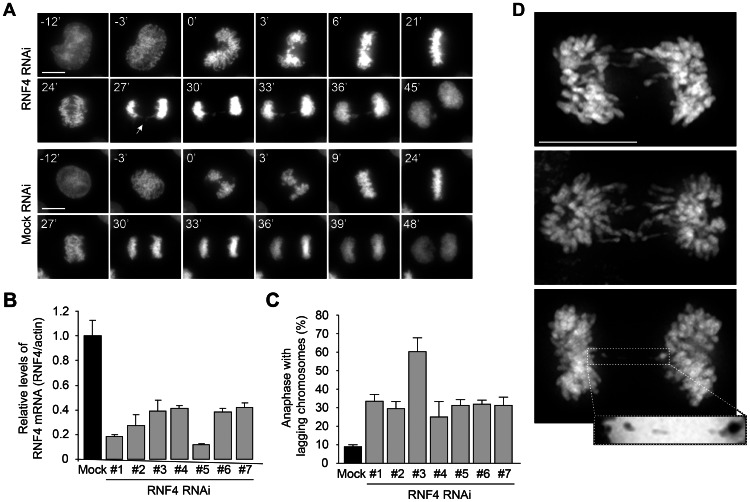
RNF4 depletion causes chromosome segregation errors. (A) Time-lapse video microscopy of H2B-EYFP HeLa cells transfected with RNF4 siRNA or mock. Arrow indicates a chromosome bridge. Time (minutes) is given relative to the first time frame in prometaphase. Scale bars, 10 µm. (B) RNF4 knockdown efficiency determined by reverse transcription qPCR. cDNA was prepared from H2B-EYFP HeLa cells transfected with siRNAs targeting RNF4 or mock siRNA. mRNA levels of *RNF4* and *β-ACTIN* were analysed by qPCR. Graph represents a single experiment, showing three technical replicates (± s.d.). (C) Quantification of chromosome segregation defects of H2B-EYFP HeLa cells, as shown in (A), transfected with different siRNAs as indicated. Graph represents the average of two independent experiments per siRNA (± s.d.) and at least 72 cells per siRNA. (D) Three examples of anaphase cells with lagging chromosomes in fixed HeLa cells transfected with RNF4 siRNA oligos. Inset (negative stain) shows a persistent chromatin bridge. Scale bars, 10 µm.

The changes in spindle morphology and elongation dynamics in *slx5/8* mutants indicate a defect during anaphase. Since the microtubule stability itself is unaffected, it suggests that this phenotype is more likely a response to a defect during chromosome segregation. The formation of fish hook spindles, in combination with an increase in spindle elongation speed, suggests that *slx5*Δ and *slx8*Δ cells have an increased need for spindle pulling force in order to separate their sister chromatids during anaphase. The small size of *S. cerevisiae* does not readily allow high-resolution morphological examination of the sister chromatids during chromosome segregation to test this hypothesis. We therefore used human HeLa cells, also to investigate whether the STUbL orthologue hRNF4 has a related role in chromosome segregation. Biochemically, hRNF4 functions in a manner that is analogous to the Slx5/8 complex in *S. cerevisiae* [17], [18], but there is as yet little evidence for a role of hRNF4 in genome stability. RNF4 was depleted from H2B-EYFP expressing HeLa cells (Figure 7A and B). While there are no discernible defects in chromosome alignment, mitotic timing or mitotic checkpoint function, the frequency of lagging chromosomes in anaphase increased three- to six-fold with different siRNA oligos (Figure 7C). Though most segregation defects are minor, anaphase bridges persisting into telophase can be discerned (Figure 7A). More detailed examination in fixed cells shows lagging chromosomes in early anaphase, with persistent chromosome bridges in late anaphase (Figure 7D). The percentage of anaphases with lagging chromosomes in fixed cells resembles that observed in live cell imaging (Figure S3). The genomic instability during anaphase and telophase upon RNF4 knockdown agrees with the phenotypes of yeast *slx5*Δ and *slx8*Δ mutants. This demonstrates that Slx5/8 and hRNF4 have an evolutionarily conserved role in maintaining genome integrity during mitosis.

## Discussion

SUMO-dependent ubiquitin ligases (STUbLs) such as Slx5/8 and RNF4 are a relatively new class of modifying enzymes, special in their ability to ubiquitinate proteins that have already been modified through sumoylation [7], [8]. Key questions regarding their cellular function and mechanism of action are unanswered. The results presented here address the cellular role of Slx5/8 in particular. Previous studies have postulated a role for Slx5/8 in regulation of transcription, through contributions to silencing [22] and turnover of transcription factors [8], [23], [24]. Although the DNA microarray analyses show that loss of either *SLX5* or *SLX8* results in upregulation of numerous genes, the majority of this transcriptional response is similar to the environmental stress response [25] and is in fact also common to inactivation of several different genome integrity pathways. Although this does not completely rule out a role for Slx5/8 in regulating gene expression, an important conclusion is that under the conditions of these experiments, the majority of gene expression changes observed upon deletion of *SLX5* or *SLX8* is likely an indirect effect of genome instability-induced stress.

An important finding presented here is the location of Slx5 at centromeres, since this may provide more focus for seeking relevant *in vivo* substrates. Interestingly, RNF4 has recently been shown to regulate the turnover of the human kinetochore protein CENP-I [41]. In yeast, the kinetochore protein Ndc10 has been reported to be sumoylated and to interact with Slx5 [32]. We have already undertaken several candidate-based approaches to find *in vivo* targets, focusing on candidates with roles at centromeres, with synthetic genetic interactions with *SLX5* and *SLX8*, and that are known to be ubiquitinated and/or sumoylated. Changes in protein levels, modifications or subcellular location in *slx5*Δ and *slx8*Δ mutants were not found for any of the candidates tested, including the kinetochore proteins Ndc10, Cep3 or Bir1. The PP2A regulatory subunit *RTS1* was found to genetically interact with *SLX5/8* and aberrantly accumulates at kinetochores during metaphase in *slx5*Δ and *slx8*Δ. Rts1 is involved in different cellular pathways, including the Spindle Positioning Checkpoint (SPOC) [42]. The SPOC is important for inhibiting mitotic exit when the anaphase spindle is misaligned along the polarity axis of the yeast cell [43]. Both *slx5*Δ and *slx8*Δ display aberrant spindle elongation kinetics and fish hook spindles in anaphase, but the positioning of the anaphase spindle is normal. There is also no delay in mitotic exit. The spindle positioning defects are exclusively observed during metaphase, which makes it unlikely that SPOC activation by PP2A^Rts1^ explains the anaphase defects of *slx5*Δ and *slx8*Δ. A more likely hypothesis is that the centromeric accumulation of PP2A^Rts1^, which is partly dependent on Sgo1, involves the tension sensing pathway. Sgo1 senses whether cells have established correct kinetochore-microtubule interactions and regulates their progression into mitosis. Cells with unattached sister chromatids lack tension at the centromeres, leading to activation of the spindle assembly checkpoint and inhibition of entry into anaphase [44]. Rts1 is recruited to the centromere by Sgo1 to protect centromeric sister chromatid cohesion [38]. Possibly, *slx5*Δ and *slx8*Δ activate the tension sensing pathway to prevent cleavage of cohesion and thereby arrest in mitosis. Indeed, observation of the Rts1 foci in *slx5*Δ and *slx8*Δ metaphase cells reveal that there is a large variability in the distance between the centromeric Rts1 foci of the sister chromatids (Figure 4C), which suggests that there is loss of centromere tension. The underlying cause that potentially triggers the tension checkpoint in *slx5*Δ and *slx8*Δ is still unclear. Slx5/8 themselves are unlikely to take part as mitotic checkpoint components, since they do not show genetic interactions with *MAD2* and are insensitive to benomyl treatment.

Several studies point to a role for Slx5/8 in the DNA damage response [5], [6], [11], [12], [16]. Loss of Slx5/8 function is thought to predominantly affect DNA replication, as this process greatly depends on accurate repair of DNA lesions that naturally occur during replication. It is therefore striking to see that the *slx5*Δ and *slx8*Δ mutant phenotypes revealed here, such as aberrant spindles and Rts1 retention, are exclusively observed during mitosis. High-resolution morphological examination of HeLa cells after RNF4 knockdown reveals the presence of lagging chromosomes and chromosome bridges, indicating that DNA damage arises during mitosis. It is unclear whether chromosome bridges also form in *slx5*Δ and *slx8*Δ. However, the altered kinetics of spindle elongation in *slx5*Δ and *slx8*Δ is suggestive of a defective separation of sister chromatids, reminiscent of chromosome bridges upon RNF4 knockdown. The source of DNA damage in *slx5*Δ and *slx8*Δ may therefore actually be the impaired segregation of chromosomes rather than defective repair of DNA replication-associated damage. We do not rule out that the origin of the defects may lie in S-phase. For instance, defective DNA decatenation during DNA replication may go unnoticed until the DNA is physically pulled apart during mitosis, ultimately leading to chromosome breaks [45]. Given the synthetic lethal genetic interactions of *SLX5/8* with members of the RecQ family of DNA helicases [5], Slx5/8 may contribute to DNA decatenation, which may lead to chromosome segregation errors and DNA breaks. Moreover, the *slx5*Δ and *slx8*Δ mutants arrest in metaphase, which clearly indicates a cellular defect that precedes the separation of sister chromatids. Previously reported roles of Slx5/8 in DNA repair and replication may therefore be directly linked to the mitotic defects observed in this study.

It is also possible that defects in DNA repair or replication and the mitotic defects represent distinct functions of Slx5/8. Whereas ChIP analysis shows that Slx5 resides at centromeres, localisation studies using fluorescence microscopy show a predominantly diffuse nuclear localisation with occasional occurrence of subnuclear foci that do not strictly colocalise with kinetochores. The foci likely represent DNA replication and repair centres [16], which would agree with distinct functions for Slx5/8 in various cellular processes. Loss of Slx5/8 function also results in general accumulation of SUMO-conjugated protein species [7], [8], suggesting that Slx5/8 targets multiple substrates for proteasomal degradation, rather than controlling a single substrate or pathway. The lack of Slx5 and Slx8 colocalisation at centromeres is unexpected given that Slx5/8 is thought to function as a heterodimeric complex [7], [8]. It suggests that only Slx5 is stably associated with the centromere, where it may function independently of Slx8 or serve to recruit Slx8 in a transient manner. We favour the latter hypothesis, based on the complete overlap of mitotic phenotypes of *slx5*Δ and *slx8*Δ mutants. Regulatory control of proteins through sumoylation is well-established to be important for several (nuclear) processes, including transcription, DNA repair and chromosome organisation [3]. The results presented here will therefore also aid future studies aimed at identification of relevant *in vivo* substrates of Slx5/8 and RNF4, by focusing on the centromere-specific location and mitotic defects reported here.

## Materials and Methods

### Yeast Strains and Media

All strains are isogenic with S288c. Yeast strains and their genotypes are listed in Table S2. Deletion strains used for microarray expression profiling were from the *Saccharomyces* Genome Deletion library (Open Biosystems; Euroscarf) and are in the genetic background of the wt parental strain BY4742. *SLX5* and *SLX8* deletion mutants were generated by PCR-based gene disruption using pFA6a deletion cassettes [46]. Single and double deletion mutants used for assaying synthetic genetic interactions were created by PCR-based gene disruption of *SLX5* or *SLX8* in the heterozygote diploid deletion strains *MAD2*/*mad2*Δ, *RTS1*/*rts1*Δ and *SGO1*/*sgo1*Δ (BY4743; Open Biosystems), followed by tetrad dissection of sporulated diploids using standard genetic techniques. We noted that spores derived from *SGO1*/*sgo1*Δ had a strong reduction in viability and aberrant segregation of the mutant alleles. Accurate gene disruption and absence of wt alleles were confirmed by PCR. Slx5-GFP and Slx8-GFP strains were constructed by C-terminal genomic integration of a pFA6a-GFP-His3MX6 cassette [46]. All epitope- or fluorescent-tagged strains exhibited wt growth with the exception of Slx5-GFP. All attempts to fuse *SLX5* to a variety of tags, either C- or N-terminally, resulted in strains with slow growth. All other epitope-tagged strains exhibited wt growth. Rts1-GFP was obtained from the GFP-tagged yeast collection [47]. Nnf1-mCherry was constructed by replacing the GFP tag from *NNF1-GFP*::His3MX6 [47] for mCherry::KanMX4. Ndc10-GFP, Cep3-GFP and Bir1-GFP strains were a kind gift of B. Montpetit [32]. Spc42-GFP (YYB3283) and GFP-Tub1 (YYB2327) are derived from previously described strains [48], [49]. The *ndc10-1* mutation was described previously [31]. Coloured colony strains were generated by backcrossing *slx5*Δ and *slx8*Δ (BY4742) twice in the genetic background of strain YYB3085 [50]. Cir^+^ yeast strains were cured of the 2 µm plasmid by overexpression of *FLP1* from the pBIS-Gal-KFLP-TRP1 and pBIS-Gal-KFLP-URA1 plasmids, and selection of Cir^0^ strains as described previously [51]. The loss of native 2 µm plasmids was monitored by PCR, using *REP1* amplifying primers (Table S3). Experiments were performed in synthetic complete (SC) or yeast extract-peptone-dextrose (YPD) media (US Biologicals) containing 2% glucose.

### Gene Expression Profiling

Microarray expression profiling was performed as described previously [52]. In brief, mutant and wt strains were grown at 30°C in SC media with 2% glucose and harvested in early mid-log phase. Dual-channel 70-mer oligonucleotide arrays were employed with a common reference wt RNA. All steps after RNA isolation were automated using robotic liquid handlers. After quality control, normalisation and dye-bias correction [53], statistical analysis was performed for each mutant versus a collection of 200 wt cultures. The reported FC is an average of four replicate mutant gene expression profiles versus the average of all wts. Fifty-eight genes that showed stochastic changes in wt profiles (wt variable genes) [54] were excluded from further downstream analyses. Clustering of the microarray expression profiles was performed using an unsupervised hierarchical cosine correlation, based on all significant genes (FC >1.7, *p*<0.05), excluding wt variable genes and deleted genes. The data is visualised using JavaTreeview [55] and GeneSpring (Agilent) software. Microarray data has been deposited in the public data repositories ArrayExpress and GEO under accession numbers E-TABM-1221 and GSE33929. The data are also available in flat-file from http://www.holstegelab.nl/publications/slx5_slx8.

### Functional Enrichment Analyses

Enrichment analysis of GO-terms [56] and transcription factor binding sites [27] was performed on all significant genes in *slx5*Δ and *slx8*Δ (FC >1.7, *p*<0.05), excluding wt variable genes and deleted genes. The background gene population was set to 6,182 (the number of genes represented on the microarray) and *p* values are Bonferroni-corrected for multiple testing. Comparison with the general stress response is performed by a Pearson correlation analysis of the average *slx5*Δ and *slx8*Δ profile with a 30-minute heat shock condition [25].

### ChIP-chip Analysis

ChIP-chip was performed essentially as described previously [57], with minor modifications. Cells were grown in 500 ml SC medium to mid-log phase at 30°C. For analysis of the temperature sensitive *ndc10-1* mutant, cells were grown overnight at 25°C and subsequently for 6 hours at either 25°C or 37°C. Cells were crosslinked with 1% formaldehyde for 20 min at room temperature (RT). Glycine (300 mM) was added for 5 min at RT. Cells were harvested by centrifugation for 5 min at 4000 rpm at 4°C. The cell pellet was washed twice with cold TBS pH 7.5 (150 mM NaCl, 10 mM Tris), once with cold FA lysis buffer (50 mM HEPES-KOH pH 7.5, 150 mM NaCl, 1 mM EDTA, 1% Triton X-100, 0.1% sodium deoxycholate, 0.1% SDS), and resuspended in 1.5 ml FA lysis buffer complemented with Complete Protease Inhibitor Cocktail (Roche). Cells were disrupted with the Disrupter Genie (Scientific Industries) using 0.5 ml zirconia beads (BioSpec Products Inc; ∅ 0.5 mm) at 4°C. The cell lysate was centrifuged 2 min at 4000 rpm at 4°C. The supernatant was centrifuged 15 min at 14,000 rpm at 4°C to collect the chromatin. The chromatin pellet was washed 30 min in 1.5 ml FA lysis buffer at 4°C, resuspended in 1.5 ml FA lysis buffer, and sonicated (Bioruptor, Diagenode: 10 cycles, 30 sec on/off, medium setting) to an average DNA fragment size of ∼400 bp. The lysate was centrifuged 20 min at 14,000 rpm at 4°C after which the supernatant (chromatin extract; CE) was collected for ChIP. ChIPs were performed by incubating 200 µl chromatin extract and 125 µg BSA to 20 µl Protein G-Agarose beads (Roche), coupled to rabbit polyclonal αGFP antibodies, for 2 h at RT. In parallel, mock ChIPs (no antibody) were performed on the same extracts. The beads were washed twice with 0.5 ml FA lysis buffer, twice in wash buffer 1 (FA lysis buffer, 500 mM NaCl), twice in wash buffer 2 (10 mM Tris pH, 0.25 mM LiCl, 0.5% Nonidet P-40, 0.5% sodium deoxycholate, 1 mM EDTA), and once in TE 10/1 (10 mM Tris pH 8, 1 mM EDTA). The beads were eluted twice in 50 µl TE 1% SDS (10 mM Tris pH 8, 1 mM EDTA, 1% SDS) for 10 min at 65°C. ChIP and input (20 µl CE) samples were incubated overnight in 100 µl TE 1% SDS and 10 µg ribonuclease A (Sigma) to reverse the formaldehyde cross-links. Samples were incubated with 400 µg proteinase K (Roche) for 2 hours at 37°C. For ChIP-chip, the proteinase K step was preceded by shrimp alkaline phosphatase (SAP) treatment by adding 1 ul of SAP (Roche) for 2 hours at 37°C. DNA was extracted with phenol-chloroform-isoamylalcohol (Sigma) and cleaned on PCR purification columns (Qiagen). Input and ChIP DNA was amplified using a robotically automated double-round T7 RNA polymerase-based amplification procedure [57]. Cy5-labelled ChIP samples were hybridised with cy3-labelled input DNA to a high-resolution 44K 4-pack yeast array (Agilent Technologies). The microarray data was quantified and normalised using a density lowess-normalisation algorithm [58]. ChIP/input and mock/input binding ratios were mapped to the ENSEMBL yeast genome EF 3 (February, 2011).

### ChIP Quantitative Real-time PCR

Non-amplified input and ChIP DNA were analysed by qPCR using the iQ SYBR Green Supermix (Bio-Rad) in a CFX96 Real-Time PCR detection system (Bio-Rad). The PCR program was 95°C/10 min, 2 cycles of 95°C/15 sec, 50°C/30 sec, 72°C/30 sec and 45 cycles of 95°C/15 sec, 58°C/30 sec, 72°C/30 sec, followed by a melting curve to check for primer specificity. Primer sequences are listed in Table S3. Binding ratios at centromeres are based on ΔCt-values (Ct ChIP/Ct input) and are presented as fold occupancy over the control gene *POL1.*

### Flow Cytometry

Cells were grown to early mid-log phase and either harvested directly as asynchronous cell population or synchronised in G1-phase by α-factor treatment (5 µg/ml; Zymo Research). Cells were released after 2.5 h in pre-warmed SC medium and harvested at 30 minute intervals. Cells (OD_600_ 1.0) were washed twice in 1 ml FACS buffer (200 mM Tris, 20 mM EDTA), resuspended in 100 µl ribonuclease A (1 mg/ml in FACS buffer; Sigma) and incubated for 2 h at 37°C at 800 rpm. Cells were washed in phosphate-buffered saline (PBS) and stained in 100 µl propidium iodide (50 µg/ml in PBS; Molecular Probes) for 1 h at RT. Sample volume was increased to 1 ml with PBS and sonicated for 10 sec at 25% amplitude (Hielscher UP200S). DNA content was quantified by flow cytometry (FACSCalibur) and analysed using CellQuest 5.2.

### Chromosome Loss Assay

Strains were grown in selective SC media lacking uracil for maintenance of the reporter chromosome and plated to single colonies on nonselective YPD plates. Colonies were allowed to grow for four days at 30°C. Red colour development was stimulated by incubating the plates one week at 4°C. The frequency of chromosome missegregation was quantified by colony half-sector analysis [59].

### Yeast Live Cell Imaging

Cells were grown asynchronously in SC medium to early mid-log phase at 30°C. Cells were transferred to a pre-warmed 8-well chambered glass-bottom Lab-TEK slide (Nunc) and covered with pre-warmed solid SC medium (5% agar). Cells were imaged on a DeltaVision RT system (Applied Precision), equipped with a heated chamber at 30°C, using a 100×/1.42-numerical aperture (NA) PlanApoN objective (Olympus). Images were acquired using Softworx software for deconvolution and are maximum intensity projections of all Z planes stacked at 0.3 µm distance. Time-lapse video microscopy was performed by acquiring Z-stacks at 2 minute intervals for 2–3 hours. Images are processed in ImageJ and Adobe Photoshop CS2. Spindle length was quantified in ImageJ and defined as the distance between two spindle pole bodies (Spc42-GFP) from pixel to pixel with the highest intensity using Z-stack maximum intensity projections. Only cells for which a complete mitosis was captured were included in the analyses. Measurements were started 20 minutes before entry into anaphase and continued for maximally one hour. Metaphase cells are scored to have mispositioned spindles if the spindle showed one or more dislocation events from the bud neck into the bud or mother cell. Spindle oscillation at the bud neck without full dislocation was scored as normal. Spindle length in metaphase cells was measured 2–4 minutes before entry into anaphase. Cells displaying a bended spindle for at least two time frames were scored as having a fish hook spindles. Maximum spindle length in anaphase cells is measured in the last time frame before spindle shortening. Spindle elongation speed was measured in mid-anaphase from 4 to 12 minutes after start of anaphase.

### Tissue Culture, Transfections and Treatments

HeLa cells and HeLa cells stably expressing H2B-EYFP were grown in DMEM supplemented with 9% FBS and pen/strep (50 µg/ml). Asynchronous cells were transfected twice with 40 nM siRNA (Table S4) using HiPerfect (Qiagen). Following the first transfection, cells were treated with 2 mM thymidine (Sigma) for 24 h. Subsequently, cells were transfected a second time and released into regular culture medium for 10 h. Cells were then treated with thymidine for 24 h and subsequently released into regular culture medium. For immunofluorescence microscopy, cells were fixed 12 h after the second release. RNF4 knockdown efficiency was measured by reverse transcription qPCR. Total rRNA was extracted using the RNeasy kit (Qiagen) including a DNase treatment step. Total RNA (250 ng) was used for cDNA synthesis (SuperScript II, Invitrogen). Expression of *RNF4* and *β-ACTIN* was analysed by qPCR (Table S3) and normalised against a standard reference cDNA from untreated H2B-EYFP HeLa cells.

### Immunofluorescence Microscopy and Live Cell Imaging of HeLa Cells

Cells, plated on 12-mm coverslips, were fixed in 3.7% Shandon Zinc Formal-Fixx (Thermo Scientific) for 10 min and permeabilised for 15 min with 0.5% Triton X-100 in PBS and washed with 0.1% Triton X-100 in PBS. Coverslips were washed and submerged in PBS containing DAPI, then washed again and mounted using ProLong antifade (Molecular Probes). Image acquisition was done using a DeltaVision RT system with a 60×/1.40NA UPlanSApo objective (Olympus) for acquiring images and SoftWorx software for deconvolution and projections. Images are maximum intensity projections of deconvolved stacks. For live cell imaging, cells were plated in eight-well chambered glass-bottomed slides (LabTek), transfected, and imaged in a heated chamber (37°C and 5% CO2) using a 60×/1.40NA UPlanSApo objective on an Olympus IX-81 microscope, controlled by Cell-M software (Olympus). Sixteen-bit yellow fluorescent images were acquired every 3 minutes using a Hamamatsu ORCA-ER camera. Images of H2B-EYFP were maximum intensity projections of all Z-planes and were processed using Cell-M software.

## Supporting Information

Figure S1 Live cell fluorescence microscopy of wt, *slx5*Δ and *slx8*Δ. *Slx5*Δ and *slx8*Δ have longer anaphase spindles and normal localisation of kinetochore components Ndc10, Cep3 and Bir1 at the centromeres and along the mitotic spindle. Scale bars, 5 µm.
(TIF)

Figure S2 *Slx5*Δ and *slx8*Δ have a mitotic delay that cannot be relieved by deletion of *MAD2*. (A) Cumulative frequency graph of the duration of G1- to M-phase. Calculation is based on the time from spindle duplication in G1-phase to spindle pole body separation in anaphase, as measured by time-lapse video microscopy of cells (n = 100) expressing Spc42-GFP. (B) Cumulative frequency graph of the duration of anaphase. Calculation is based on the time from start of spindle elongation to spindle depolymerisation, as measured by time-lapse video microscopy of cells (n = 100) expressing Tub1-GFP. (C) Growth rate assay of cells spotted in five-fold serial dilutions on YPD plates. Images are after two days growth at 30°C. (D) Cell cycle progression of synchronized cells. DNA content was measured by flow cytometry at 30 minute intervals over a period of four hours after release from α-factor arrest in G1-phase. Arrows indicate cell populations with 1N (G1-phase) and 2N (G2/M-phase) DNA content.
(TIF)

Figure S3 Quantification of chromosome segregation defects in fixed HeLa cells. Graph represents the average of two independent experiments (± s.d.) and at least 110 cells per siRNA. Anaphases with chromosome segregation defects other than lagging chromosomes were infrequent in both mock and RNF4 knockdown situation and not considered for these analyses.
(TIF)

Figure S4 Comparison of *slx5/8* phenotypes in the presence (Cir^+^) or absence (Cir^0^) of the 2 µm plasmid. (A) PCR verification of the loss of the 2 µm plasmid in three independent strains of wt, *slx5*Δ and *slx8*Δ. (B) Flow cytometric profiles of asynchronous populations of Cir^+^ and Cir^0^ wt, *slx5*Δ and *slx8*Δ strains. Cell population with a >2N DNA content, indicated in red, is quantified (± s.d., n = 3). (C) Growth rate of yeast cultures in liquid YPD media. Relative growth rate (mutant/wt) was quantified during mid-log phase (± s.d., n = 3). (D) Quantification of spindle length in wt Cir^0^ and *slx8*Δ Cir^0^ strains, expressing Spc42-GFP. Spindle length is quantified as described in Figure 6E. (E) Comparison of spindle phenotypes in Cir^+^ and Cir^0^ wt and *slx8*Δ strains during metaphase and anaphase.
(TIF)

Table S1 GO and TFBS enrichment analyses. Gene Ontology (GO) and transcription factor binding site (TFBS) enrichment analyses of differentially expressed genes (mutant vs. wt, FC >1.7, *p*<0.05). The dataset is provided as separate Excel file.
(XLS)

Table S2 Yeast strains.
(DOCX)

Table S3 Primer sequences.
(DOCX)

Table S4 SiRNA sequences.
(DOCX)
