# Supplementary figures and images for: Centromere Binding and a Conserved Role in Chromosome Stability for SUMO-Dependent Ubiquitin Ligases
Source: PLoS One. 2013 Jun 13;8(6):e65628. doi: 10.1371/journal.pone.0065628 (PMC3681975; doi:10.1371/journal.pone.0065628)

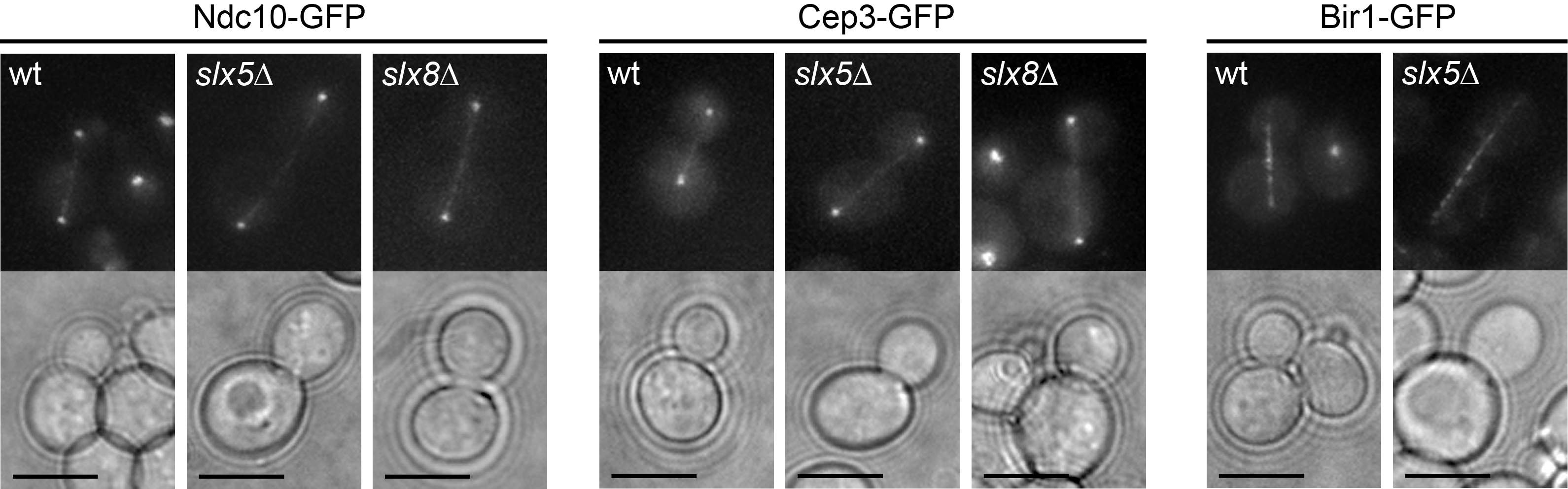

Supplement: Figure S1 — Live cell fluorescence microscopy of wt, slx5Δ and slx8Δ. Slx5Δ and slx8Δ have longer anaphase spindles and normal localisation of kinetochore components Ndc10, Cep3 and Bir1 at the centromeres and along the mitotic spindle. Scale bars, 5 µm. (TIF) [file pone.0065628.s001.tif]

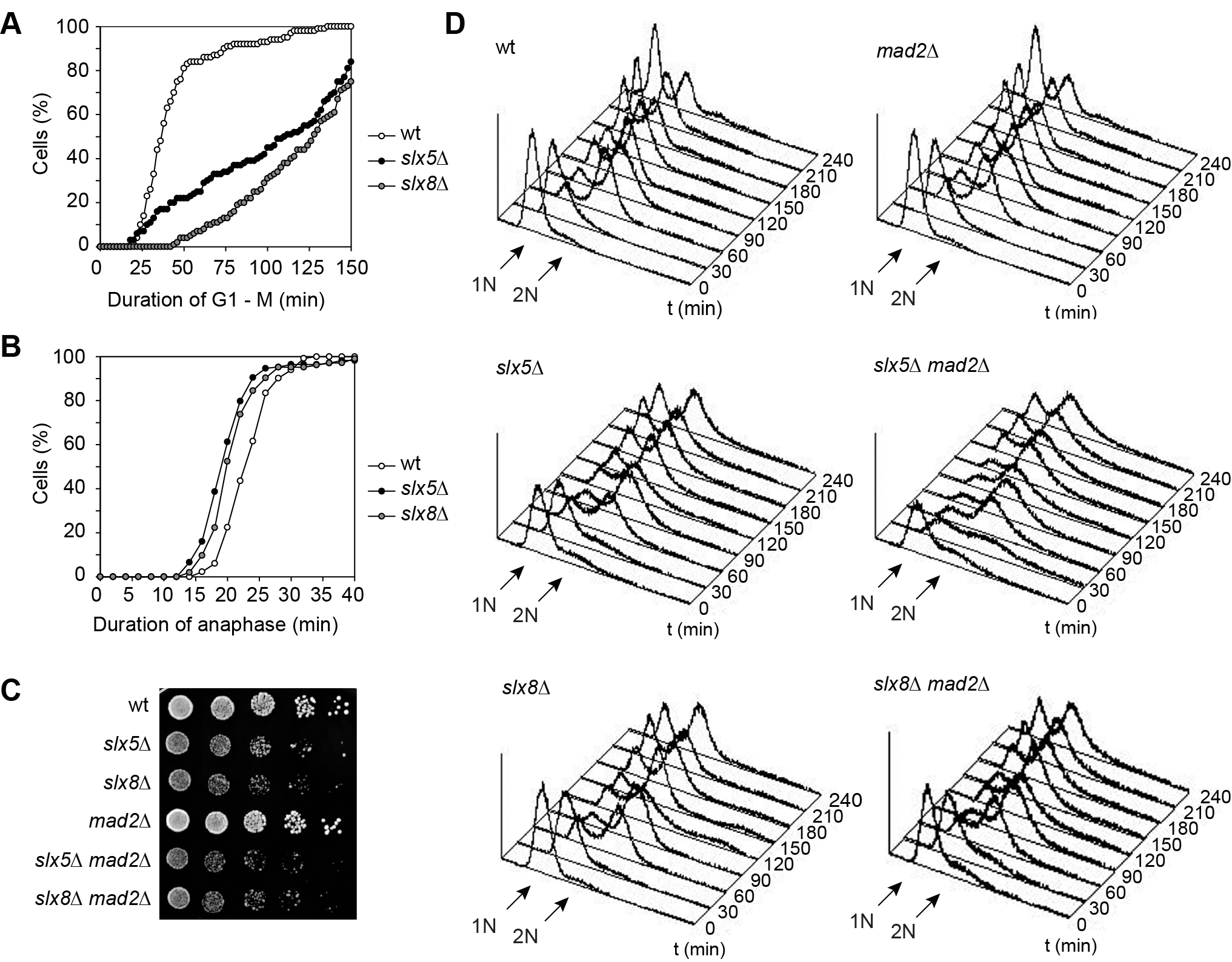

Supplement: Figure S2 — Slx5Δ and slx8Δ have a mitotic delay that cannot be relieved by deletion of MAD2. (A) Cumulative frequency graph of the duration of G1- to M-phase. Calculation is based on the time from spindle duplication in G1-phase to spindle pole body separation in anaphase, as measured by time-lapse video microscopy of cells (n = 100) expressing Spc42-GFP. (B) Cumulative frequency graph of the duration of anaphase. Calculation is based on the time from start of spindle elongation to spindle depolymerisation, as measured by time-lapse video microscopy of cells (n = 100) expressing Tub1-GFP. (C) Growth rate assay of cells spotted in five-fold serial dilutions on YPD plates. Images are after two days growth at 30°C. (D) Cell cycle progression of synchronized cells. DNA content was measured by flow cytometry at 30 minute intervals over a period of four hours after release from α-factor arrest in G1-phase. Arrows indicate cell populations with 1N (G1-phase) and 2N (G2/M-phase) DNA content. (TIF) [file pone.0065628.s002.tif]

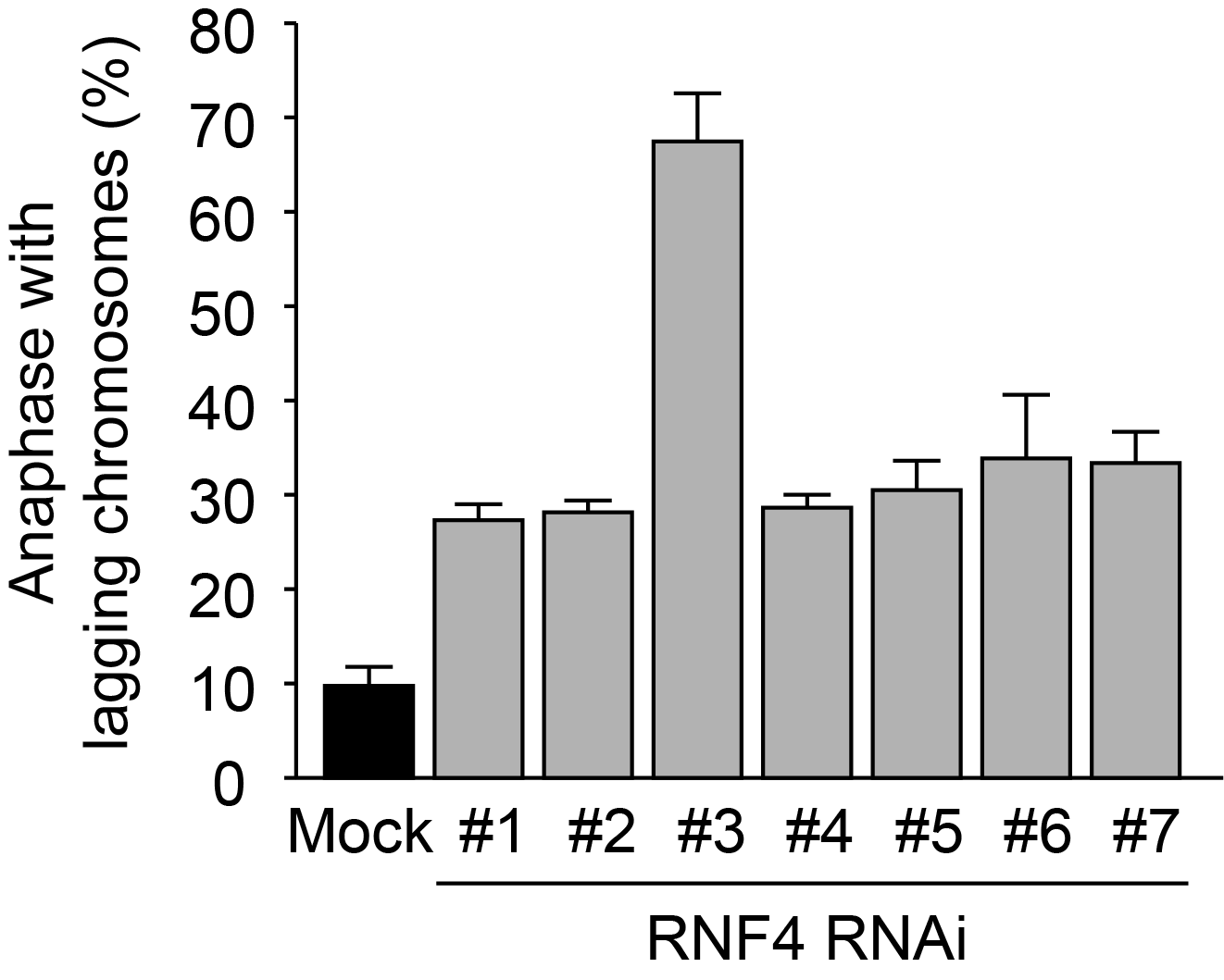

Supplement: Figure S3 — Quantification of chromosome segregation defects in fixed HeLa cells. Graph represents the average of two independent experiments (± s.d.) and at least 110 cells per siRNA. Anaphases with chromosome segregation defects other than lagging chromosomes were infrequent in both mock and RNF4 knockdown situation and not considered for these analyses. (TIF) [file pone.0065628.s003.tif]

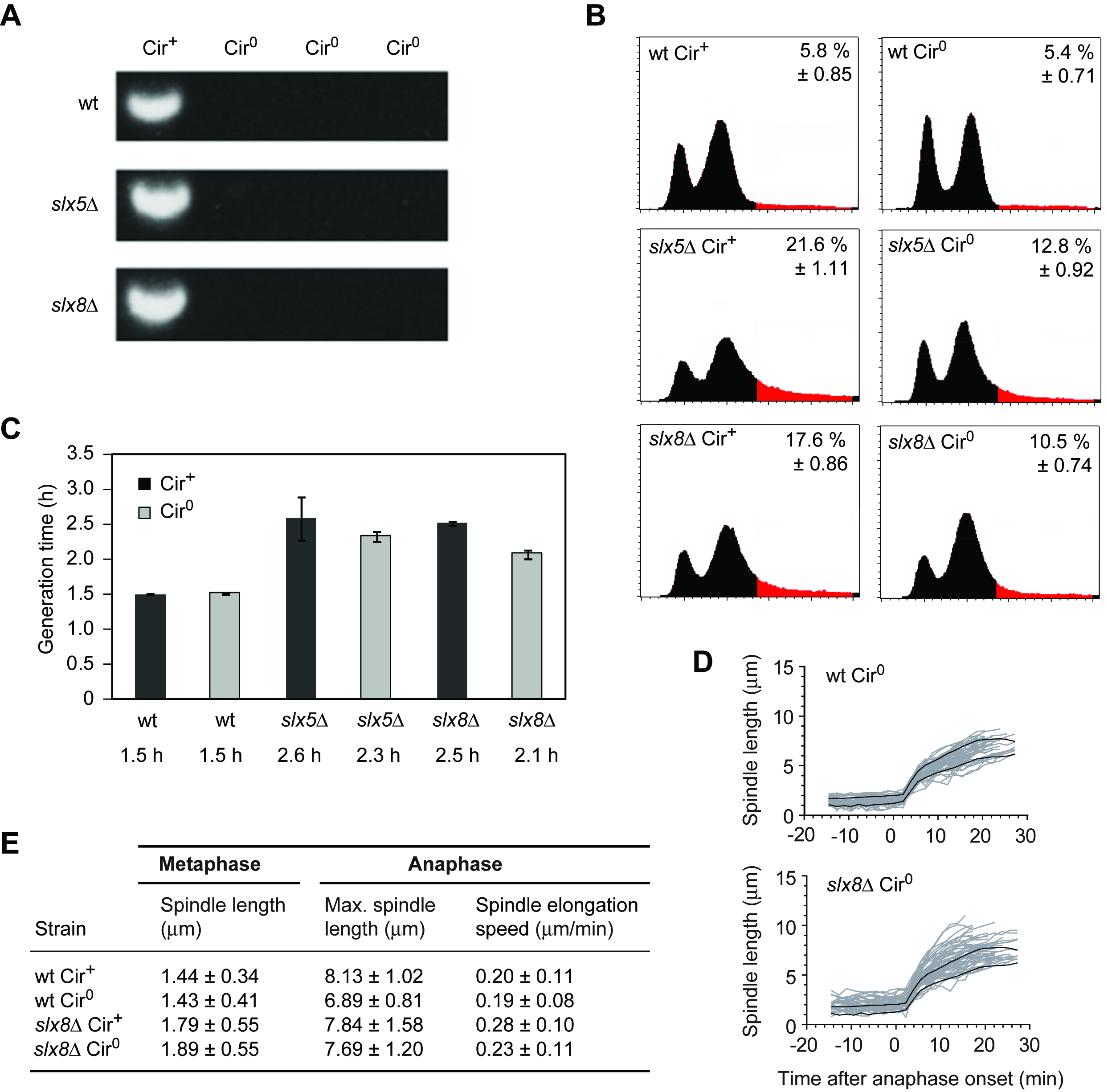

Supplement: Figure S4 — Comparison of slx5/8 phenotypes in the presence (Cir+) or absence (Cir0) of the 2 µm plasmid. (A) PCR verification of the loss of the 2 µm plasmid in three independent strains of wt, slx5Δ and slx8Δ. (B) Flow cytometric profiles of asynchronous populations of Cir+ and Cir0 wt, slx5Δ and slx8Δ strains. Cell population with a >2N DNA content, indicated in red, is quantified (± s.d., n = 3). (C) Growth rate of yeast cultures in liquid YPD media. Relative growth rate (mutant/wt) was quantified during mid-log phase (± s.d., n = 3). (D) Quantification of spindle length in wt Cir0 and slx8Δ Cir0 strains, expressing Spc42-GFP. Spindle length is quantified as described in Figure 6E. (E) Comparison of spindle phenotypes in Cir+ and Cir0 wt and slx8Δ strains during metaphase and anaphase. (TIF) [file pone.0065628.s004.tif]
