# Supplementary material for: Centromere Binding and a Conserved Role in Chromosome Stability for SUMO-Dependent Ubiquitin Ligases
Source: PLoS One. 2013 Jun 13;8(6):e65628. doi: 10.1371/journal.pone.0065628 (PMC3681975; doi:10.1371/journal.pone.0065628)
Supplement: Table S2 — Yeast strains. (DOCX) [file pone.0065628.s006.docx]

**Table S2. Yeast strains.**

| **Name** | **Genotype** | **Source** |
| --- | --- | --- |
| BY4741 | MATa *his3-∆1 leu2-∆0 lys2-∆0 ura3-∆0* | Open Biosystems |
| BY4742 | MATα *his3-∆1 leu2-∆0 met15-∆0 ura3-∆0* | Open Biosystems |
| TLS001 | MATα *his3-∆1 leu2-∆0 met15-∆0 ura3-∆0 slx5::KanMX6* | This study |
| YTM006 | MATα *his3-∆1 leu2-∆0 met15-∆0 ura3-∆0 slx8::KanMX6* | This study |
| YFR036W | MATα *his3-∆1 leu2-∆0 met15-∆0 ura3-∆0 cdc26::KanMX4* | Open Biosystems |
| YEL061C | MATα *his3-∆1 leu2-∆0 met15-∆0 ura3-∆0 cin8::KanMX4* | Euroscarf |
| YCR086W | MATα *his3-∆1 leu2-∆0 met15-∆0 ura3-∆0 csm1::KanMX4* | Open Biosystems |
| YPR135W | MATα *his3-∆1 leu2-∆0 met15-∆0 ura3-∆0 ctf4::KanMX4* | Euroscarf |
| YMR078C | MATα *his3-∆1 leu2-∆0 met15-∆0 ura3-∆0 ctf18::KanMX4* | Open Biosystems |
| YPL018W | MATα *his3-∆1 leu2-∆0 met15-∆0 ura3-∆0 ctf19::KanMX4* | Open Biosystems |
| YDR318W | MATα *his3-∆1 leu2-∆0 met15-∆0 ura3-∆0 mcm21::KanMX4* | Open Biosystems |
| YDL188C | MATα *his3-∆1 leu2-∆0 met15-∆0 ura3-∆0 pph22::KanMX4* | Open Biosystems |
| YCR066W | MATα *his3-∆1 leu2-∆0 met15-∆0 ura3-∆0 rad18::KanMX4* | Open Biosystems |
| YNL250W | MATα *his3-∆1 leu2-∆0 met15-∆0 ura3-∆0 rad50::KanMX4* | Open Biosystems |
| YML032C | MATα *his3-∆1 leu2-∆0 met15-∆0 ura3-∆0 rad52::KanMX4* | Open Biosystems |
| YPL024W | MATα *his3-∆1 leu2-∆0 met15-∆0 ura3-∆0 rmi1::KanMX4* | Euroscarf |
| YMR190C | MATα *his3-∆1 leu2-∆0 met15-∆0 ura3-∆0 sgs1::KanMX4* | Open Biosystems |
| YGL060W | MATα *his3-∆1 leu2-∆0 met15-∆0 ura3-∆0 ybp2::KanMX4* | Open Biosystems |
| YLP522 | MATα *his3-∆1 leu2-∆0 ura3-∆0 slx5::His3MX6* | This study |
| YLP524 | MATa *his3-∆1 leu2-∆0 met15-∆0 ura3-∆0 slx5::His3MX6* | This study |
| YLP534 | MATa *his3-∆1 leu2-∆0 lys2-∆0 ura3-∆0 slx5::NatMX6* | This study |
| YLP536 | MATα *his3-∆1 leu2-∆0 met15-∆0 ura3-∆0 slx5::NatMX6* | This study |
| YLP525 | MATa *his3-∆1 leu2-∆0 met15-∆0 ura3-∆0 slx8::His3MX6* | This study |
| YLP527 | MATα *his3-∆1 leu2-∆0 lys2-∆0 met15-∆0 ura3-∆0 slx8::His3MX6* | This study |
| YLP504 | MATα *his3-∆1 leu2-∆0 ura3-∆0 slx8::NatMX6* | This study |
| YLP505 | MATa *his3-∆1 leu2-∆0 ura3-∆0 slx8::NatMX6* | This study |
| YLP538 | MATa *his3-∆1 leu2-∆0 met15-∆0 ura3-∆0 rts1::KanMX4* | This study |
| YLP540 | MATα *his3-∆1 leu2-∆0 lys2-∆0 ura3-∆0 rts1::KanMX4* | This study |
| YLP543 | MATa *his3-∆1 leu2-∆0 met15-∆0 ura3-∆0 rts1::KanMX4 slx5::NatMX6* | This study |
| YLP545 | MATα *his3-∆1 leu2-∆0 lys2-∆0 ura3-∆0 rts1::KanMX4 slx5::NatMX6* | This study |
| YLP547 | MATa *his3-∆1 leu2-∆0 met15-∆0 lys2-∆0 ura3-∆0 rts1::KanMX4 slx8::His3MX6* | This study |
| YLP550 | MATα *his3-∆1 leu2-∆0 met15-∆0 lys2-∆0 ura3-∆0 rts1::KanMX4 slx8::His3MX6* | This study |
| YLP530 | MATα *his3-∆1 leu2-∆0 met15-∆0 lys2-∆0 ura3-∆0 sgo1::KanMX4* | This study |
| YLP531 | MATα *his3-∆1 leu2-∆0 met15-∆0 ura3-∆0 sgo1::KanMX4 slx5::NatMX6* | This study |
| YLP533 | MATα *his3-∆1 leu2-∆0 lys2-∆0 ura3-∆0 sgo1::KanMX4 slx8::NatMX6* | This study |
| YLP512 | MATα *his3-∆1 leu2-∆0 met15-∆0 ura3-∆0 mad2::KanMX4* | This study |
| YLP514 | MATa *his3-∆1 leu2-∆0 met15-∆0 ura3-∆0 mad2::KanMX4* | This study |
| YLP516 | MATα *his3-∆1 leu2-∆0 met15-∆0 ura3-∆0 mad2::KanMX4 slx5::His3MX6* | This study |
| YLP518 | MATa *his3-∆1 leu2-∆0 met15-∆0 ura3-∆0 mad2::KanMX4 slx5::His3MX6* | This study |
| YLP519 | MATα *his3-∆1 leu2-∆0 met15-∆0 ura3-∆0 mad2::KanMX4 slx8::His3MX6* | This study |
| YLP521 | MATa *his3-∆1 leu2-∆0 met15-∆0 ura3-∆0 mad2::KanMX4 slx8::His3MX6* | This study |
| YLP033 | MATα *his3-∆1 leu2-∆0 met15-∆0 ura3-∆0 SLX8-GFP::His3MX6* | This study |
| YLP034 | MATα *his3-∆1 leu2-∆0 met15-∆0 ura3-∆0 SLX5-GFP::His3MX6* | This study |
| YYB2168 | MATa *ade2-101 his3-Δ200 leu2 lys2-801 trp1-Δ63 ura3-52 ndc10-1* | This study |
| YLP437 | MATa *ade2-101 his3-Δ200 leu2 lys2-801 trp1-Δ63 ura3-52 SLX5-GFP::His3MX6 ndc10-1* | This study |
| YLP438 | MATα *his3-∆1 leu2-∆0 met15-∆0 ura3-∆0 SLX5-GFP::His3MX6 slx8::NatMX6* | This study |
| YLP439 | MATα *his3-∆1 leu2-∆0 met15-∆0 ura3-∆0 SLX8-GFP::His3MX6 slx5::NatMX6* | This study |
| YYB3085 | MATa *ade2-101 his3-∆200 leu2-∆1 lys2-801 trp1-∆63 ura3-52 CFIII (CEN3.L.YPH278) URA3 SUP11* | Spencer *et al*., 1990 |
| YLP440 | MATa *ade2-101 his3-∆200 leu2-∆1 lys2-801 trp1-∆63 ura3-52 CFIII (CEN3.L.YPH278) URA3 SUP11 slx5::His3MX6* | This study |
| YLP441 | MATa *ade2-101 his3-∆200 leu2-∆1 lys2-801 trp1-∆63 ura3-52 CFIII (CEN3.L.YPH278) URA3 SUP11 slx8::His3MX6* | This study |
| YBM556 | MATα *ura3-52 ade2-101 trp1-∆63 his3-∆200 leu2-∆1 NDC10-GFP::His3MX6* | Montpetit *et al*., 2006 |
| YLP578 | MATa *his3-∆200 leu2-∆1 lys2-801 ura3-52 NDC10-GFP::His3MX6 slx5::KanMX6* | This study |
| YLP580 | MATa *his3-∆200 leu2-∆1 lys2-801 trp1-∆63 ura3-52 NDC10-GFP::His3MX6 slx8::NatMX6* | This study |
| YPH1819 | MATa *ade2-101 his3-∆200 leu2-∆1 lys2-801 trp1-∆63 ura3-52 CEP3-GFP::His3MX6* | Montpetit *et al*., 2006 |
| YLP583 | MATa *his3-∆200 leu2-∆1 lys2-801 ura3-52 CEP3-GFP::His3MX6 slx5::KanMX6* | This study |
| YLP585 | MATα *his3-∆200 leu2-∆1 lys2-801 trp1-∆63 ura3-52 CEP3-GFP::His3MX6 slx8::NatMX6* | This study |
| YPH1821 | MATa *ade2-101 his3-∆200 leu2-∆1 lys2-801 trp1-∆63 ura3-52 BIR1-GFP::His3MX6* | Montpetit *et al*., 2006 |
| YLP553 | MATa *his3-∆200 leu2-∆1 lys2-801 ura3-52 BIR1-GFP::His3MX6 slx5::KanMX6* | This study |
| YYB3820 | MATα *his3-∆1 leu2-∆0 met15-∆0 ura3-∆0 Nnf1-mCherry::KanMX4* | This study |
| YLP225 | MATa *his3-∆1 leu2-∆0 met15-∆0 ura3-∆0 Nnf1-mCherry::KanMX4 SLX5-GFP::His3MX6* | This study |
| YLP160 | MATa *his3-∆1 leu2-∆0 met15-∆0 ura3-∆0 RTS1-GFP::His3MX6* | Huh *et al*., 2003 |
| YLP161 | MATa *his3-∆1 leu2-∆0 ura3-∆0 RTS1-GFP::His3MX6 slx5::KanMX6* | This study |
| YLP162 | MATα *his3-∆1 leu2-∆0 lys2-∆0 ura3-∆0 RTS1-GFP::His3MX6 slx5::KanMX6* | This study |
| YLP163 | MATa *his3-∆1 leu2-∆0 met15-∆0 ura3-∆0 RTS1-GFP::His3MX6 slx8::KanMX6* | This study |
| YLP165 | MATα *his3-∆1 leu2-∆0 lys2-∆0 ura3-∆0 RTS1-GFP::His3MX6 slx8::KanMX6* | This study |
| YLP422 | MATa *his3-∆1 leu2-∆0 met15-∆0 ura3-∆0 RTS1-GFP::His3MX6 sgo1::KanMX6* | This study |
| YLP426 | MATa *his3-∆1 leu2-∆0 met15-∆0 ura3-∆0 RTS1-GFP::His3MX6 sgo1::KanMX6 slx5::NatMX6* | This study |
| YLP427 | MATα *his3-∆1 leu2-∆0 ura3-∆0 Nnf1-mCherry::KanMX4 RTS1-GFP::His3MX6* | This study |
| YLP429 | MATa *his3-∆1 leu2-∆0 ura3-∆0 Nnf1-mCherry::KanMX4 RTS1-GFP::His3MX6 slx5::KanMX6 mad2::URA3* | This study |
| YYB2327 | MATα *ura3-52 lys2-801 ade2-101 trp1-∆63 his3-∆200 leu2-∆1 GFP-TUB1::URA3* | Grava *et al*., 2006 |
| YLP144 | MATa *ura3-52 lys2-801 ade2-101 trp1-∆63 his3-∆200 leu2-∆1 GFP-TUB1::URA3 slx8::KanMX6* | This study |
| YLP146 | MATα *ura3-52 lys2-801 ade2-101 trp1-∆63 his3-∆200 leu2-∆1 GFP-TUB1::URA3* *slx5::His3MX6* | This study |
| YYB3283 | MATa *ura3-52 lys2-801 ade2-101 trp1-∆63 his3-∆200 leu2-∆1 SPC42-GFP::hphNT1* | Neurohr *et al*., 2011 |
| YLP076 | MATa *ura3-52 lys2-801 ade2-101 trp1-∆63 his3-∆200 leu2-∆1 SPC42-GFP::hphNT1 slx5::KanMX6* | This study |
| YLP077 | MATa *ura3-52 lys2-801 ade2-101 trp1-∆63 his3-∆200 leu2-∆1 SPC42-GFP::hphNT1 slx8::KanMX6* | This study |
| YTM101 | MATa *ura3-52 lys2-801 ade2-101 trp1-∆63 his3-∆200 leu2-∆1 SPC42-GFP::hphNT1 Cir^0^* | This study |
| YTM102 | MATa *ura3-52 lys2-801 ade2-101 trp1-∆63 his3-∆200 leu2-∆1 SPC42-GFP::hphNT1 slx5::KanMX6 Cir^0^* | This study |
| YTM101 | MATa *ura3-52 lys2-801 ade2-101 trp1-∆63 his3-∆200 leu2-∆1 SPC42-GFP::hphNT1 slx8::KanMX6 Cir^0^* | This study |
| YTM107 | MATa *his3-∆1 leu2-∆0 met15-∆0 ura3-∆0 RTS1-GFP::His3MX6 Cir^0^* | This study |
| YTM108 | MATa *his3-∆1 leu2-∆0 ura3-∆0 RTS1-GFP::His3MX6 slx5::KanMX6 Cir^0^* | This study |
| YTM110 | MATa *his3-∆1 leu2-∆0 met15-∆0 ura3-∆0 RTS1-GFP::His3MX6 slx8::KanMX6 Cir^0^* | This study |
