# Supplementary material for: Centromere Binding and a Conserved Role in Chromosome Stability for SUMO-Dependent Ubiquitin Ligases
Source: PLoS One. 2013 Jun 13;8(6):e65628. doi: 10.1371/journal.pone.0065628 (PMC3681975; doi:10.1371/journal.pone.0065628)
Supplement: Table S3 — Primer sequences. (DOCX) [file pone.0065628.s007.docx]

**Table S3. Primer sequences.**

| **Name** | **Direction** | **Sequence (5’ – 3’)** |
| --- | --- | --- |
| CEN1 | Forward | AACTTCAAAACCTTTTATGTAA |
|  | Reverse | AGGCGCTTGAAATGAAAGCTCCG |
| CEN2 | Forward | CTTGAGCAAATTGATCCTACATAAT |
|  | Reverse | GCAAGAAATATATTGATACTTC |
| CEN5 | Forward | CTATGAAACATCAAATTAATCA |
|  | Reverse | CGGAAATCTAATACTGCTACAA |
| POL1 | Forward | TGCACCAGTTAATTCTAAAAAGGCA |
|  | Reverse | AAAACACCCTGATCCACCTCTGAA |
| RNF4 | Forward | CTCGTGGAAACTGCTGGAGATGAAATTG |
|  | Reverse | ATTCCTCCTTGGTCTTCTTCTTTCGTCAAC |
| β-Actin | Forward | GACATGGAGAAAATCTGGCA |
|  | Reverse | AATGTCACGCACGATTTCCC |
| Rep1 | Forward | ACAGCGCTGATATACAATG |
|  | Reverse | CTGTCGGCTATTATCTCCG |
| Rep1A | Forward | GCCAGAGGATGGCGAACC |
|  | Reverse | GCTCGCGTTGCATTTTCG |
