# Supplementary material for: Centromere Binding and a Conserved Role in Chromosome Stability for SUMO-Dependent Ubiquitin Ligases
Source: PLoS One. 2013 Jun 13;8(6):e65628. doi: 10.1371/journal.pone.0065628 (PMC3681975; doi:10.1371/journal.pone.0065628)
Supplement: Table S4 — SiRNA sequences. (DOCX) [file pone.0065628.s008.docx]

**Table S4. SiRNA sequences.**

| **Name** | **#** | **Sequence (5’ – 3’)** |
| --- | --- | --- |
| Luciferase | Mock | CGTACGCGGAATACTTCGA |
| RNF4-2 | #1 | GAATGGACGTCTCATCGTTTT |
| RNF4-5 | #2 | CCCTGTTTCCTAAGAACGAAA |
| RNF4-8 | #3 | AAGACTGTTTCGAAACCAACA |
| RNF4-D7 | #4 | GCTAATACTTGCCCAACTT |
| RNF4-D8 | #5 | GAATGGACGTCTCATCGTT |
| RNF4-D9 | #6 | GACAGAGACGTATATGTGA |
| RNF4-D10 | #7 | GCAATAAATTCTAGACAAG |
